# Fungal strain matters: colony growth and bioactivity of the European medicinal polypores *Fomes fomentarius, Fomitopsis pinicola* and *Piptoporus betulinus*

**DOI:** 10.1186/s13568-014-0093-0

**Published:** 2015-01-24

**Authors:** Philipp Dresch, Maria Nives D´Aguanno, Katharina Rosam, Ulrike Grienke, Judith Maria Rollinger, Ursula Peintner

**Affiliations:** Institute of Microbiology, University of Innsbruck, Technikerstraße 25, 6020 Innsbruck, Austria; Department of Life Sciences, Università degli Studi di Siena, Via Mattioli 4, 53100 Siena, Italy; Department of Pharmacognosy, Faculty of Life Sciences, University of Vienna, Althanstraße 14, 1090 Vienna, Austria

**Keywords:** Fungal strain selection, Temperature optimum, Wood-rotting fungi, Antimicrobial activity of fungal extracts, Phylogeny

## Abstract

Polypores have been applied in traditional Chinese medicine up to the present day, and are becoming more and more popular worldwide. They show a wide range of bioactivities including anti-cancer, anti-inflammatory, antiviral and immuno-enhancing effects. Their secondary metabolites have been the focus of many studies, but the importance of fungal strain for bioactivity and metabolite production has not been investigated so far for these *Basidiomycetes*. Therefore, we screened several strains from three medicinal polypore species from traditional European medicine: *Fomes fomentarius, Fomitopsis pinicola* and *Piptoporus betulinus.* A total of 22 strains were compared concerning their growth rates, optimum growth temperatures, as well as antimicrobial and antifungal properties of ethanolic fruit body extracts. The morphological identification of strains was confirmed based on rDNA ITS phylogenetic analyses. Our results showed that species delimitation is critical due to the presence of several distinct lineages, e.g. within the *Fomes fomentarius* species complex. Fungal strains within one lineage showed distinct differences in optimum growth temperatures, in secondary metabolite production, and accordingly, in their bioactivities. In general, *F. pinicola* and *P. betulinus* extracts exerted distinct antibiotic activities against *Bacillus subtilis* and *Staphylococcus aureus* at minimum inhibitory concentrations (MIC) ranging from 31-125 μg mL^−1^; The antifungal activities of all three polypores against *Aspergillus flavus, A. fumigatus, Absidia orchidis* and *Candida krusei* were often strain-specific, ranging from 125-1000 μg mL^−1^. Our results highlight that a reliable species identification, followed by an extensive screening for a ‘best strain’ is an essential prerequisite for the proper identification of bioactive material.

**Electronic supplementary material:**

The online version of this article (doi:10.1186/s13568-014-0093-0) contains supplementary material, which is available to authorized users.

## Introduction

Polypores are a diverse group of *Agaricomycetes* (*Basidiomycota*) with tough poroid hymenophores. These fungi have been extensively applied in traditional Chinese medicine (TCM) up to the present day (Chang 2000; Chang and Wasser 2012; Hobbs 1995; Ying et al. 1987) and are becoming more and more popular also in other parts of the world where they are used as a source for medicinal compounds and therapeutic adjuvants, or as health food supplements. They show a wide range of bioactivities including anti-cancer, anti-inflammatory, antiviral and immuno-enhancing effects (Grienke et al. 2014; Molitoris 2005; Paterson 2006). The large number of scientific studies focusing on their bioactivity (Khatun et al. 2011; Lo and Wasser 2011; Xu et al. 2010; Zhao 2013; Zhong and Xiao 2009; Zhou et al. 2011), secondary metabolite production (Hwang et al. 2013; You et al. 2013) and genomics (Floudas et al. 2012; Liu et al. 2012) is therefore not surprising.

Secondary metabolites of medicinal polypores have been the focus of many studies (Grienke et al. 2014; Zjawiony 2004), but the importance of fungal strain for bioactivity and metabolite production has not been investigated so far. Strain-specific differences in bioactivities (on the function of macrophages) were detected for different strains of the Ascomycete *Ophiocordyceps sinensis,* whose fruit bodies are highly praised as traditional remedy (Meng et al. 2013). Distinct strain differences are also known for anamorphic ascomycetes, e.g. for the secondary metabolite production of *Fusarium avenaceum*, where substrate is playing an additional important role in the regulation of secondary metabolite biosynthesis (Sørensen and Giese 2013).

Fungal medicinal polypore species belonging to the same genus differ clearly in their metabolite profile (Hseu et al. 1996; Lv et al. 2012).

Species delimitation of polypores was long based upon macro-morphological and ecological characters. However, these classical morphological species concepts have proven to be too wide for many important medicinal polypore species, e.g. for *Ganoderma lucidum, G. applanatum*, or *Laetiporus sulphureus* (Banik et al. 2010; Banik et al. 2012; Hseu et al. 1996, Ota et al. 2009). Polypore species, described upon morphology as generalists on several hosts, have often turned out to consist of multiple phylogenetic (cryptic) lineages, and the description of new species if often necessary within such species complexes (Skaven Seierstad et al. 2013). This makes comparison of studies working on metabolites from medicinal polypores forming a morphological species complex especially critical and difficult. It is therefore essential to document, from which habitat and substrate the fruit body was obtained, to deposit a voucher in a public mycological collection and a culture in a public culture collection, in order to check or to re-identify the fungus. Moreover, it is also recommended to sequence barcoding regions of the investigated fungal material, in order to allow for a later verification of the identification of the fungus.

A recent study on the secondary metabolites of the medicinal polypore *Laricifomes officinalis* was carried out in an exemplary way by also including rDNA ITS sequences of the used strain (Hwang et al. 2013). But screening for optimal strains is rarely carried out, and strain differences within polypore species are usually not taken into account, although it is well known that a large variety of existing alleles are promoting outcrossing of polypores; genetic exchange in dikaryons by somatic recombination creates additional new combinations of alleles (Peabody et al. 2000). This implies that ecologically relevant genetic variation permanently occurs within dikaryotic fungal individuals, allowing for evolutionary adaptation and thus for the evolution of physiologically distinct strains.

To study polypore species with known bioactivities allows for an easy and straightforward comparison of different strains from one species, e.g. for their antimicrobial properties or their metabolic fingerprint. We therefore investigated strain-specific differences of three medicinal polypore species of the traditional European medicine. The applications of these polpores in traditional medicine, and knowledge on their metabolite profile have been extensively reviewed by Grienke et al. (2014).

When using defined commercially available culture strains, interpretation of the results is often difficult due to lack of information, e.g. on the environment, substrate, or on the fruit body morphology. We therefore used newly isolated strains, and extensively documented their habitat, substrate, and the morphology of fruit bodies. This also allowed for a comparison of strains based on both, bioactivities of fruit body extracts and the physiological properties of cultures *in vitro*.

Physiological properties are often used for species delimitation because they reflect genetic distance: *Fomes* strains belonging to different species have different physiological properties, like colony growth responses to temperature (McCormick et al. 2013). The question whether also strains within one species, e.g. *F. fomentarius,* differ in growth responses to temperature was not addressed so far.

The main aim of this work was to study the influence of fungal strain (isolate) of three selected, medicinal polypores on their antimicrobial bioactivity and physiological properties. The bioactivity was assessed for ethanolic extracts from the same fruit bodies, which had been used for isolation of mycelial cultures. Bioactivity was tested against potentially pathogenic fungi and bacteria. Physiological properties of mycelial cultures (colony growth rate and optimum temperature) were investigated under standard *in vitro* conditions. All polypore strains used were characterized by rDNA ITS sequences, and phylogenetic analyses were carried out to verify species identification. The potential influence of geographic provenance and substrate on strain evolution and properties are discussed.

## Material and methods

### Organism collection, identification, culture

*F. fomentarius, F. pinicola,* and *P. betulinus* fruit bodies were mainly collected in Tyrol, Austria in 2013. Fruit body vouchers were deposited in the Mycological Collection of the Herbarium Innsbruck (IB), cultures were deposited in the Jena Microbial Resource Collection (JMRC), sequences were deposited in GenBank. Voucher numbers, GenBank accession numbers, JMRC collection numbers, plant hosts, as well as habitat information are given in Table 1.

**Table 1:** **Overview of polypore strains used in this study: information on voucher ID, Genbank accession number, JMRC strain number, natural substrate (host), provenance, altitude and optimum growth temperature for each isolate**

**Species**	**Voucher**	**GenBank**	**JMRC**	**Substrate**	**Country**	**Altitude [m]**	**Opt. Temp. [°C]**
*F. fomentarius*	20130011	KM360125	SF:11850	*P. abies*	Austria	817	25
*F. fomentarius*	20130016	KM360126	SF:11851	*P. abies*	Austria	817	25
*F. fomentarius*	20130019	KM360127	SF:11852	*F. sylvatica*	Austria	1300	25
*F. fomentarius*	20130022	KM360128	SF:11853	*P. abies*	Austria	820	25
*F. fomentarius*	20130033	KM360129	SF:11854	*Q. pubescens*	Italy	500	32
*F. fomentarius*	P74908	KM396269	SF:11850	*Betula sp.*	Austria	-	n.t.
*F. pinicola*	20130010	KM360130	SF:11855	*P. abies*	Austria	1720	25
*F. pinicola*	20130013	KM360131	SF:11856	*P. abies*	Austria	1729	25
*F. pinicola*	20130015	KM360132	SF:11857	*P. abies*	Austria	1732	25
*F. pinicola*	20130018	KM360133	SF:11858	*P. abies*	Austria	1420	25
*F. pinicola*	20130021	KM360134	SF:11859	*P. abies*	Austria	812	25
*F. pinicola*	20130024	KM360135	*-*	*P. abies*	Austria	787	n.t.
*F. pinicola*	20130026	KM360136	SF:11860	*A. incana*	Austria	812	32
*F. pinicola*	20130030	KM360137	SF:11861	*L. decidua*	Austria	1870	32
*F. pinicola*	20130034	KM360138	SF:11862	*P. abies*	Italy	1182	25
*F. pinicola*	20130037	-	*-*	*A. alba*	Italy	1200	n.t.
*F. pinicola*	20130040	KM360139	SF:11863	*P. abies*	Austria	812	25
*F. pinicola*	20130042	KM360140	SF:11864	*P. abies*	Austria	850	25
*F. pinicola*	20130053	-	*-*	*P. abies*	Austria	1820	n.t.
*F. pinicola*	P34908	KM396268	SF:11864	*P. abies*	Austria	-	n.t.
*P. betulinus*	20130029	KM360141	SF:11865	*Betula sp.*	Austria	817	25
*P. betulinus*	20130039	KM360142	SF:11866	*Betula sp.*	Italy	600	25
*P. betulinus*	20130055	KM411430	SF:11867	*Betula sp.*	Austria	998	25

Sterile techniques were used to obtain cultures from the context tissue. Small pieces of context tissue (2.0 mm^3^) were excised from each basidiome, plated on 2-3 % w/v malt extract agar plates (MEA Art. Nr. X976.2 from Roth Karlsruhe Germany) and incubated for one to three weeks at a temperature of 20°C. Cultures were checked regularly for contaminations. Mycelial plugs of 1-3 mm in diameter were taken from the edge of the mycelium and transferred to new plates, to establish pure cultures and further carrying out growth experiments.

The tissue cultures and stock cultures are maintained at the Institute of Microbiology, University of Innsbruck, Austria. For cryopreservation small parts of well-growing cultures were overlaid with 10 % glycerol (Sigma), incubated for 1 h at room temperature, plugged out and stored at −80°C. Isolates were also stored on malt extract agar (MEA) slants at 4°C. The partial sequences of ITS rRNA genes allowed for the identification of the strains. Sequence data are available from GenBank (Table 1). Phylogenetic analyses were used to confirm the morphological identification of all strains and to test for sequence divergence.

### Colony growth temperature experiments

Five isolates of *F. fomentarius,* and 11 isolates of *F. pinicola* and 3 isolates of *P. betulinus* were grown on plates containing 25 mL MEA. After 14 d, four mycelia plugs (5 mm diam.) were taken 1 cm from the leading edge of the colony and transferred to the middle of plates of 9 cm diam. containing 25 mL MEA. Plates were randomly placed into a plastic box, and incubated at six different temperatures (10, 20, 25, 30, 32 and 37°C). Mean colony diameter (mm), minus the 5 mm plug, was measured after 2, 5, 7 and 10 days. The results are expressed as means ± standard deviations of three parallel cultures. Significant differences between colony growth response to temperature on different days were identified using one-way analysis of variance (ANOVA) (p < 0.05) followed by post-hoc Tukey’s HSD test (α = 0.05), using R version 3.1.0 (2014-04-10) for Windows (R Development Core Team, 2014).

### DNA amplification and sequence analysis

Molecular identification of isolated mycelia was performed using sequence data of the ITS regions of the ribosomal DNA. Total genomic DNA was isolated from 100 μg of fungal matter (one-month-old mycelial cultures or fresh basidiome) by DNeasy® Plant Mini Kit (QIAGEN, Germany) according to the manufacturer's instructions and then eluted in 50 μl of sterile water. ITS-1, 5.8S rRNA and ITS-2 regions were amplified in a 50 μl volume reaction containing 1-10 ng of genomic DNA, using the primers pair ITS1 and ITS4 in a T gradient Thermal Cycler (primus 96; Peqlab, Germany) according to Peintner et al. (2001). PCR products were sequenced by Microsynth AG (Switzerland) with the primer ITS4. Sequences were analysed using the Sequencher® software (version 5.2.3; Gene Codes, Ann Arbor, MI, USA).

As a first step, Blast searches were conducted in GenBank (http://ncbi.nlm.nih.gov), and closely related sequences were downloaded. Only a small part of identical sequences were downloaded in order to cover geographical range and substrate preferences. Alignment and phylogenetic analyses were carried out with MEGA 5.2 (Tamura et al. 2011).

Sequences were aligned and manually adjusted in MEGA 5.2 for each genus. To evaluate branch robustness of trees, parsimony-based bootstrap analyses were applied. ML analyses were conducted based on the best ML model estimated. Bootstrap analyses were conducted using 1000 replications, SPR search method, search-level 5. The alignment and phylograms have been submitted to TreeBASE (http://treebase.org).

### Ethanolic extraction of fruit bodies

For each strain 1 g of dried and milled fruit body was placed in a glass centrifugation tube and immersed in 30 mL 96% EtOH. The tube was covered with aluminium foil and sonicated for 20 min at room temperature. After centrifuging for 5 min at 3 500 rpm the supernatant was filtered through a Pasteur pipette stuffed with cotton wool. After washing of the fungal material with 10 mL of 96% EtOH, the combined extract was evaporated to dryness. The dry ethanolic extracts were dissolved in 96% EtOH to a final concentration of 10 mg mL^−1^. These stock solutions were stored at −20°C.

### HPLC analysis of fungal extracts

HPLC analyses were carried out using a Shimadzu UFLC XR device at 40°C, a flow rate of 1.0 mL min^−1^, injection volume was 10 μL, detection wavelength 254 nm. The stationary phase was a Phenomenex® HyperClone ODS (C18) column, 120 Å, 5 μm, 150 × 4.60 mm; mobile phase A: H_2_O + 0.9% AA + 0.1% FA; mobile phase B: CH_3_CN + 0.9% AA + 0.1% FA; 0 min: 90% A to 30 min: 2% A, 30-35 min: 2% A.

### Bioactivity of fungal extracts

Screenings for antibiotic/antifungal activities of EtOH extracts were carried out in 96-well micro plates. Four bacterial strains (*Escherichia coli* ATCC 11229, *Staphylococcus aureus* ATCC 25923, *Pseudomonas aeruginosa* ATCC 27853, *Bacillus subtilis* MB37) and four potential pathogenic or toxin-producing fungal species (*Absidia orchidis, Aspergillus fumigatus, A. flavus* and *Candida krusei*) were used for quantitative bioassays. All microorganisms were tested separately: Micro plates were inoculated with one test organism, each, and used to either test five fungal extracts at 8 concentrations, or 10 fungal extracts at 4 concentrations. Negative, positive and solvent controls were included on each micro plate.

### *In vitro* antibacterial assays

Minimum inhibitory concentrations (MICs) were determined using the broth microdilution method (serial 2fold dilutions) as recommended by the Clinical Laboratory Standards Institute (CLSI 2012). The lowest concentration leading to no growth (i.e. no turbidity), as assessed by macroscopic evaluation was taken as the minimum inhibitory concentration (MIC).

Bacterial cultures were maintained on LB agar slants. Each bacterial strain was streaked out on a LB agar plate and incubated for 24 hours at 35 ± 2°C. Four to five single standing colonies were used to inoculate 20-50 mL LB in 100 mL Erlenmeyer flasks. The bacteria were allowed to grow for 6-8 hours at 35 ± 2°C and 150 rpm until a slight turbidity was obtained. The cell density was checked with a cytometer (Thomakammer). Bacteria were diluted in sterile LB to a density of 10^6^ cells mL^−1^ and vortexed thoroughly. Bacterial suspension was added to the micro plates as described below. Sterility controls for broth and flasks were included, and bacterial inoculum was always streaked out on an LB plate to check for contamination.

Sterile 96-well micro plates (columns 1-12, rows A-H) were filled with 50 μL LB. Starting from column 3 (to 10) serial two-fold dilutions of either fungal extracts or control antibiotic (tetracycline, H3-10) were made, with the highest concentration starting at 1000 μg mL^−1^ (final). One row received the same volume of solvent (96% EtOH) as introduced with the fungal extracts, while another row received deion. sterile water, to determine any possible inhibiting effect of the solvent on bacterial growth. Afterwards, 50 μL of bacterial suspension were added to each well, except those in the last two columns (11, 12) to a give final cell concentration of 5 × 10^5^ mL^−1^. Columns 11 and 12 instead received another 50 μL of sterile LB (final volume 100 μL) and worked as broth sterility controls. Columns 1 and 2 received sterile LB and bacterial inoculum and worked as growth controls.

The micro plates were sealed with parafilm® to prevent desiccation and edge effects and incubated for 20 h at 35 ± 2°C. Plates were evaluated by visually checking for growth by comparing the turbidity in the wells with that of the growth and broth control, respectively. Only plates with completely turbid growth, as well as clear broth controls were analysed.

For determination of bactericidal effects 10 μL of each test well showing growth inhibition in the primary screening were transferred into 100 μL fresh LB and incubated for 48 h. The specific concentrations were accepted as bactericidal, if no growth occurred after incubation in a concentration lower than the corresponding MIC.

No technical replicates were integrated in the primary screening in order to save as much of the restricted amount of extracts available. Therefore the lowest test concentration showing an inhibitory effect was repeated in 3 parallels. If this second test showed the same results, the results were considered as verified. If not, the test was repeated with the next highest concentration of the dilution series until the MIC could be verified. Fungal extracts themselves usually increased the turbidity of the medium (debris, pigments), therefore an extra micro plate containing only fungal extracts in LB was set up as a reference to aid visual comparison.

### *In vitro* antifungal assays

For filamentous fungi, a 96-well based micro plate approach was carried out similar to the one described by Troskie et al. (2012). *Absidia orchidis, A. flavus* and *A. fumigatus* were cultivated on potato dextrose agar (PDA) until sporulation set in (approx. one week). Spores were harvested in 3 mL Tween20 (0.1% in aqua dest.) and spore density was assessed using a cytometer (Neubauer). The spore solution was diluted in 0.5 × strength potato dextrose broth (PDB) to a density of 10^5^ spores mL^-1^. *Candida krusei* was streaked out on a PDA plate, and incubated for 24 h at 35 ± 2°C. Four to five single standing colonies were used to inoculate 20-50 mL PDB in 100 mL Erlenmeyer flasks. The yeasts were allowed to grow for 6-8 h at 35 ± 2°C and 150 rpm until a slight turbidity was obtained. The cell density was checked with a cytometer (Neubauer). Yeasts were diluted in sterile PDB to a cell density of 10^5^ mL^−1^ and 20 μL well^−1^ of this yeast inoculum were used to inoculate the micro plates.

Micro plates were filled with 70 μL sterile 1 × strength PDA. Plates were sealed in plastic bags and stored at 4°C until the assay was started. Test substance stock solutions and 0.5 x strength PDB were combined in the wells to final concentrations of 1000, 500, 250 and 125 μg mL^−1^, respectively. Cycloheximide (serial dilutions starting at 1000 μg mL^−1^) as well as the serial dilutions of 96% EtOH were integrated on changing positions, and used as negative control (AB susceptibility control) and as solvent inhibition control, respectively. Thereafter, 20 μL of spore solution were added to each well, except to the broth sterility control wells. Micro plates were incubated at 25°C for at least 48 h. Then the wells were checked for fungal growth using a stereo microscope. A subsequent protein staining of the plates with Coomassie Blue G250 was carried out as described by (Troskie et al. 2012) to allow discrimination between fungal biomass and precipitates of fruit body extracts.

*Candida krusei* micro plates were prepared based on the layout and protocol for filamentous fungi. However only liquid PDB was used, and micro plates were shaken periodically (for 5” every 30’) to avoid on-surface growth.

## Results

### Hosts, isolation, and cultivation of fungal strains

In sum, nineteen fungal strains were isolated for this study: five strains of *F. fomentarius,* 11 strains of *F. pinicola*, and three strains *of P. betulinus*. Strains were isolated from a narrow geographical area spanning a maximum of hundred kilometres, with a few control strains isolated form the southern side of the Alps, and one strain from the mediterranean area. *F. fomentarius* was isolated from *Fagus sylvatica, Picea abies* and *Quercus pubescens; F. pinicola* was isolated from *Abies alba*, *Alnus incana, Larix decidua* and *P. abies. P. betulinus* was isolated from *Betula pubescens* only*.*

### HPLC analysis of fruit body extracts

High-performance liquid chromatography (HPLC) of fruit body extracts showed that polypore strains differ in both, qualitative and quantitative secondary metabolite production. *F. fomentarius* strain IB20130019 was significantly different by showing an extremely poor production of secondary metabolites. *F. pinicola* strains showed a very high variation in the quantity of secondary metabolites produced (Additional file 1: Figure S1).

### Bioactivity of extracts

#### Bioactivity of *F. fomentarius* extracts

Fruit body extracts from all strains of *F. fomentarius* showed minimum inhibitory concentrations (MICs) of 250-500 μg mL^−1^ against *Staphylococcus aureus*, and MICs of 125-500 μg mL^−1^ against *A. fumigatus* (Ascomycota) and *A. orchidis* (*Zygomycota*) (Table 2). The only exception was strain IB20130019, which was isolated from a subalpine habitat and a *Fagus sylvatica* substrate. This strain was not bacteriostatic at concentrations ≤ 500 μg mL^−1^, nor fungistatic at ≤ 1000 μg mL^−1^ against *A. orchidis*. HPLC chromatograms confirmed that both, the quantity and quality of secondary metabolites produced by this strain IB20130019 are clearly different from secondary metabolites produced by the other strains.

**Table 2:** **Minimum inhibitory concentrations (MIC) and/or minimum bactericial concentrations (MBC) of fruit body extracts**

		***B. subtilis***		***S. aureus***		***A. flav.***	***A. fum.***	***A. orc.***	***C. kru.***
**Species**	**Voucher IB**	**MIC**	**MBC**	**MIC**	**MBC**	**MIC**	**MIC**	**MIC**	**MIC**
*F. fom.*	20130011	-	-	500*	-	-	-	500	-
*F. fom.*	20130016	-	-	250*	-	-	125	250	-
*F. fom.*	20130019	-	-	-	-	-	n.t.	-	-
*F. fom.*	20130022	-	-	250*	-	-	125	500	-
*F. fom.*	20130033	-	-	250*	-	-	125	250	1000*
*F. pin.*	20130010	63*	125	500*	-	-	125	500	-
*F. pin.*	20130013	125*	-	500*	500	-	125	500	-
*F. pin.*	20130015	63*	-	31*	125*	-	125	1000	-
*F. pin.*	20130018	63*	125	31*	31*	-	500	-	-
*F. pin.*	20130021	125*	500	63*	250*	-	500	x	-
*F. pin.*	20130024	31*	31	31*	31*	-	500	500	-
*F. pin.*	20130026	63*	63	31*	63*	1000	500	500	-
*F. pin.*	20130030	31*	31	63*	63	1000	500	500	-
*F. pin.*	20130034	31*	63	63*	125	-	x	500	n.t.
*F. pin.*	20130037	63*	500	63*	125	1000	500	500	-
*F. pin.*	20130040	31*	31	63*	125	x	125	1000	-
*F. pin.*	20130042	63*	63	125*	250	-	x	500	-
*F. pin.*	20130053	31*	31	63*	250	-	x	-	-
*P. bet.*	20130029	63*	500	31*	31*	-	x	500	n.t.
*P. bet.*	20130039	63*	500	31*	31*	-	x	1000	n.t.

Minimum inhibitory concentrations (MIC) of fruit body extracts against bacteria (*Bacillus subtilis* and *Staphylococcus aureus*), a selection of filamentous fungi, and the yeast *Candida kruseii* (*C. kru.*) (μg mL^−1^). Minimum bactericial concentrations (MBC) are provided for bacteria. Minus (−): no inhibition observed using the highest test concentration of 1000 μg mL^−1^; asterisks (*): the inhibitory effect was confirmed by 3 replicates; x: alterations in growth morphology (branching); n.t.: not tested. The results for the two gram-negative bacteria tested (*E. coli, P. aeruginosa*) are not shown in the table, because none of the tested extracts showed an inhibitory effect at ≤ 1000 μg mL^−1^. For *Trichophyton sp*., already the solvent showed an inhibitory effect at a concentration of 4.8 % EtOH. *A. flav. = Aspergillus flavus, A. fum.* = *Aspergillus fumigatus, A. orc.* = *Absidia orchidis.*

The Mediterranean isolate IB20130033, which belongs to another clade, was the only strain showing an effect against *Candida krusei* at 1000 μg mL^−1^. This strain produced significantly higher quantities of secondary metabolites than the other strains (Additional file 1: Figure S1).

#### Bioactivity of *F. pinicola* extracts

*F. pinicola* extracts showed good bacteriostatic and bactericidal bioactivities with MICs of 31-125 μg mL^−1^ against *B. subtilis* and of 31-500 μg mL^−1^ against *S. aureus.* A general antifungal activity was detected against *A. fumigatus,* and most *F. pinicola* strains had also antifungal activity against *Absidia orchidis* at 500-1000 μg mL^−1^. Three strains (IB20130026, IB20130030, IB20130037) showed an additional antifungal activity against *A. flavus*. All these latter strains are from other substrates (*Abies, Alnus, Picea, and Larix*) than *Picea abies*.

#### Bioactivity of *P. betulinus* extracts

*P. betulinus* extracts showed a good antibacterial activity against *B. subtilis* (MIC 62.5 μg mL^−1^, MBC 500 μg mL^−1^) and *S. aureus* (MIC/MBC 31 μg mL^−1^)*.* Extracts inhibited the growth of *Absidia orchidis* at 500-1000 μg mL^−1^, and caused abnormal growth of *A. fumigatus*.

### Growth rates of fungal strains

#### Colony growth and optimum temperatures of *F. fomentarius* strains

**Figure 1:**
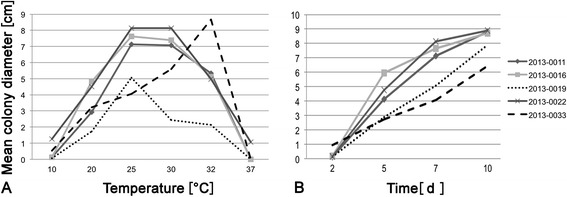
**Mean colony diameter of isolated** ***F. fomentarius*** **strains on MEA (n = 3 per strain). A**. After 7 days of incubation at different temperatures. **B**. Mean growth kinetics at 25°C. ANOVA result at 25°C (optimal growth temperature for major part of strains) p = 1.345e-05. Strains with significant differences after post hoc test: 20130019-20130011 (p = 0.006); 20130019-20130016 (p = 0.001); 20130019-20130022 (p = 0.0003); 2013-0033-20130011 (p = 0.0003); 20130033-20130016 (p = 0.00009); 20130033-20130022 (p = 0.00003).

The three *F. fomentarius* strains isolated from *Picea* (IB20130011, IB20130016, and IB20130022) showed similar colony diameters after 7 d of incubation at different temperatures, with optimal growth temperatures of 25 or 30°C, leading to a mean colony diameter of 7.0-8.1 cm (Figure 1A and Additional file 1: Table S1). The growth kinetics at 25°C (i.e. increase in colony diameter per measurement) were also similar for these strains. In comparison, the strain isolated from *F. sylvatica* (IB20130019) was growing slower on all tested temperatures, with a maximum of 5 cm colony diameter reached after 7 d incubation at 25°C (optimum temperature) and significantly lower growth kinetics (Figure 1B) This can be due to the previous growth on *Fagus* as a host, or due to the provenance from a subalpine area at 1300 m a. s. l. The strain isolated form *Q. pubescens* (IB20130033) also differed significantly from all other strains, with a relatively slow growth at 25°C and 30°C compared to the *Picea*-derived strains, and the highest measured colony diameter compared to all *Fomes* strains (8.7 ± 0.5 cm) at 32°C. (Figure 1). This strain differs by its provenance from a sub-Mediterranean area, and by growing on *Quercus* as a host from all other strains.

#### Colony growth and optimum temperatures of *F. pinicola* strains

**Figure 2:**
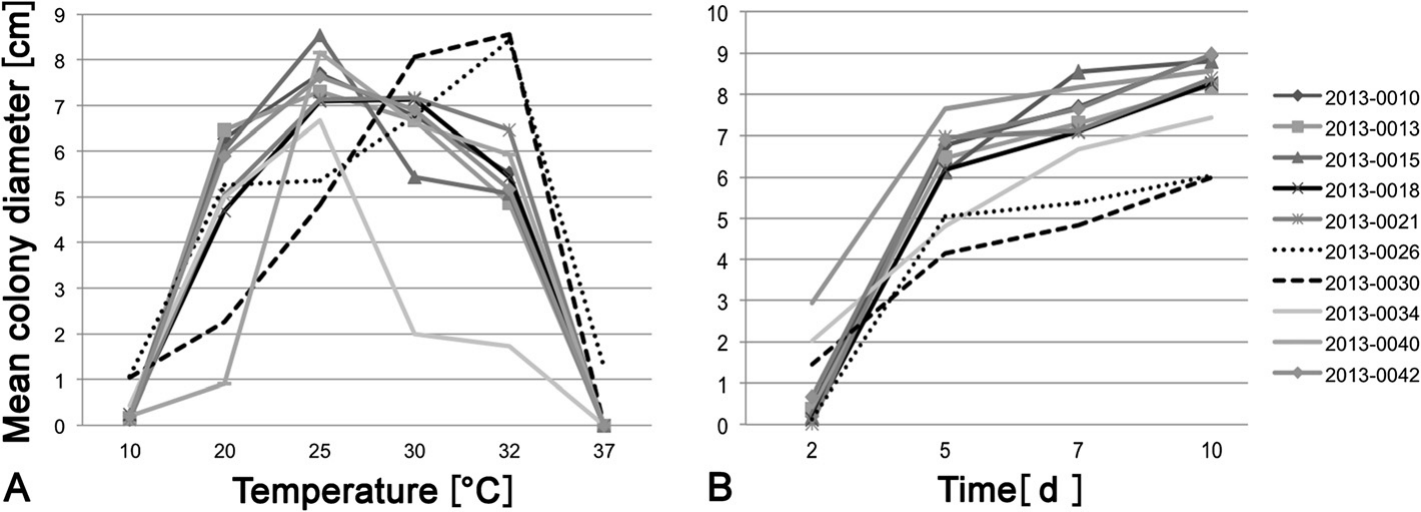
**Mean colony diameter of isolated** ***F. pinicola*** **strains on MEA (n = 3 per strain). A**. After 7 days of incubation at different temperatures. **B**. Mean growth kinetics at 25°C. ANOVA result at 25°C (optimal growth temperature) p = 0.000125. Strains with significant differences after post hoc test: 20130026-20130010 (p = 0.028); 20130026-20130015 (p = 0.002); 20130026-20130040 (p = 0.006); 20130026-20130042 (p = 0.04); 20130030-20130010 (p = 0.004); 20130030-20130013 (p = 0.018); 20130030-20130015 (p = 0.0002); 20130030-20130018 (p = 0.04); 20130030-20130021 (p = 0.03); 20130030-20130040 (p = 0.0008); 20130030-20130042 (p = 0.006).

Most *F. pinicola* strains isolated from *Picea* had an optimal growth temperature of 25°C after 7d. Only two *Picea*-derived strains (IB20130018 and IB20130021) differed by having an optimum temperature of 30°C (Figure 2A). However, for these two strains the differences in colony diameter between 25 and 30°C were not significant (Additional file 1: Table S2). *Picea* strains, although originating from the same substrate, exhibited different growth kinetics (Figure 2B and Additional file 1: Table S2). Strains from *Alnus* (IB20130026) and *Larix* (IB20130030) substrates had a strikingly different temperature optimum of 32°C and different growth kinetics (Figures 2A and 2B).

#### Colony growth and optimum temperatures of *P. betulinus* strains

**Figure 3:**
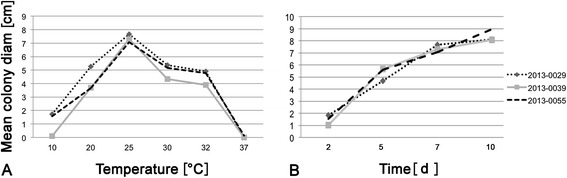
**Mean colony diameter of** ***P. betulinus*** **strains on MEA (n = 3 per strain). A**. After 7 days of incubation at different temperatures. **B**. Mean growth kinetics at 25°C.

The optimum growth temperature for the three isolated *P. betulinus* strains was at 25°C (Figure 3A and Additional file 1: Table S3), with no significant differences between strains regarding optimum temperature or growth kinetics (Figure 3B), not even for strain IB20130055, although it was isolated from another geographical region.

### Phylogenetic placement of polypore strains based on rDNA ITS sequences

#### Phylogenetic placement of *F. fomentarius* strains

**Figure 4:**
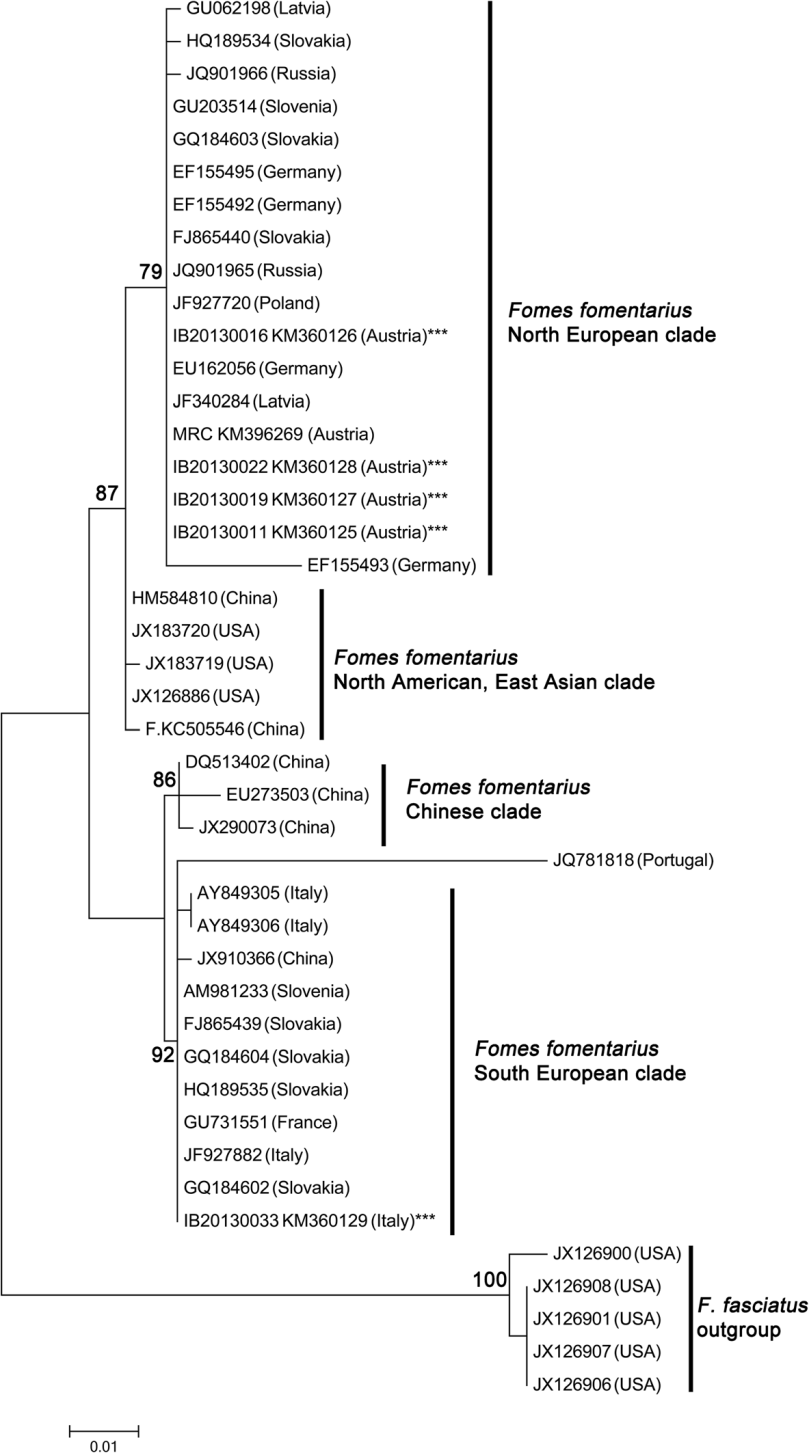
**Phylogenetic placement of** ***F. fomentarius*** **strains inferred by using the Maximum Likelihood method based on the Kimura 2-parameter model.** The tree with the highest log likelihood (-1266,9966) is shown. Initial tree(s) for the heuristic search were obtained automatically by applying Neighbor-Join and BioNJ algorithms to a matrix of pairwise distances estimated using the Maximum Composite Likelihood (MCL) approach, and then selecting the topology with superior log likelihood value. The tree is drawn to scale, with branch lengths measured in the number of substitutions per site. The analysis involved 43 nucleotide sequences. All positions with less than 95% site coverage were eliminated. That is, fewer than 5% alignment gaps, missing data, and ambiguous bases were allowed at any position. There were a total of 505 positions in the final dataset. Bootstrap values above 75 % are given (1 000 replications). Evolutionary analyses were conducted in MEGA6.

The phylogenetic analysis of *F. fomentarius* sequences generated for this study showed that the geographic origin and the substrate are important factors driving speciation in this genus of facultative opportunistic phytopathogenic *Basidiomycetes*. The phylogenetic tree allows for distinction of four well-supported clades within *F. fomentarius* (BS > 75 %). *Fomes fasciatus* was used as outgroup (Figure 4).

Our strains clustered into two clades: the four strains isolated from the alpine range fell within a clade of *F. fomentarius* sequences with origin in the Northern European countries (Russia, Poland, Latvia, Slovak Republic, Germany, Austria, Slovenia), and *F. sylvatica, Alnus* spp., *Acer negundo* and *P. abies* as substrates. This Northern European clade is sister to a clade of *F. fomentarius* from the U.S.A and China from *Betula* spp. substrates.

Our Mediterranean strain (IB20130033) was placed within another, distinct clade of *F. fomentarius* sequences originating mostly from Southern European countries (Italy, France, Portugal, Slovenia) and other substrates (*Platanus x acerifolia, Populus spp., Quercus spp.* and *Abies*). The only exceptions are *F. fomentarius* sequences from the Slovak Republic. This Southern European clade is sister to a distinct clade of *F. fomentarius* from China.

Within clade sequence divergence was small, with 0-3 base pair differences between the different strains of *F. fomentarius* from Northern European countries. Sequence divergence between the Northern and Southern European clade was 9-18 base pairs, and sequence divergence to the outgroup *F. fasciatus* was 41-62 base pairs.

#### Phylogenetic placement of *F. pinicola* strains

**Figure 5:**
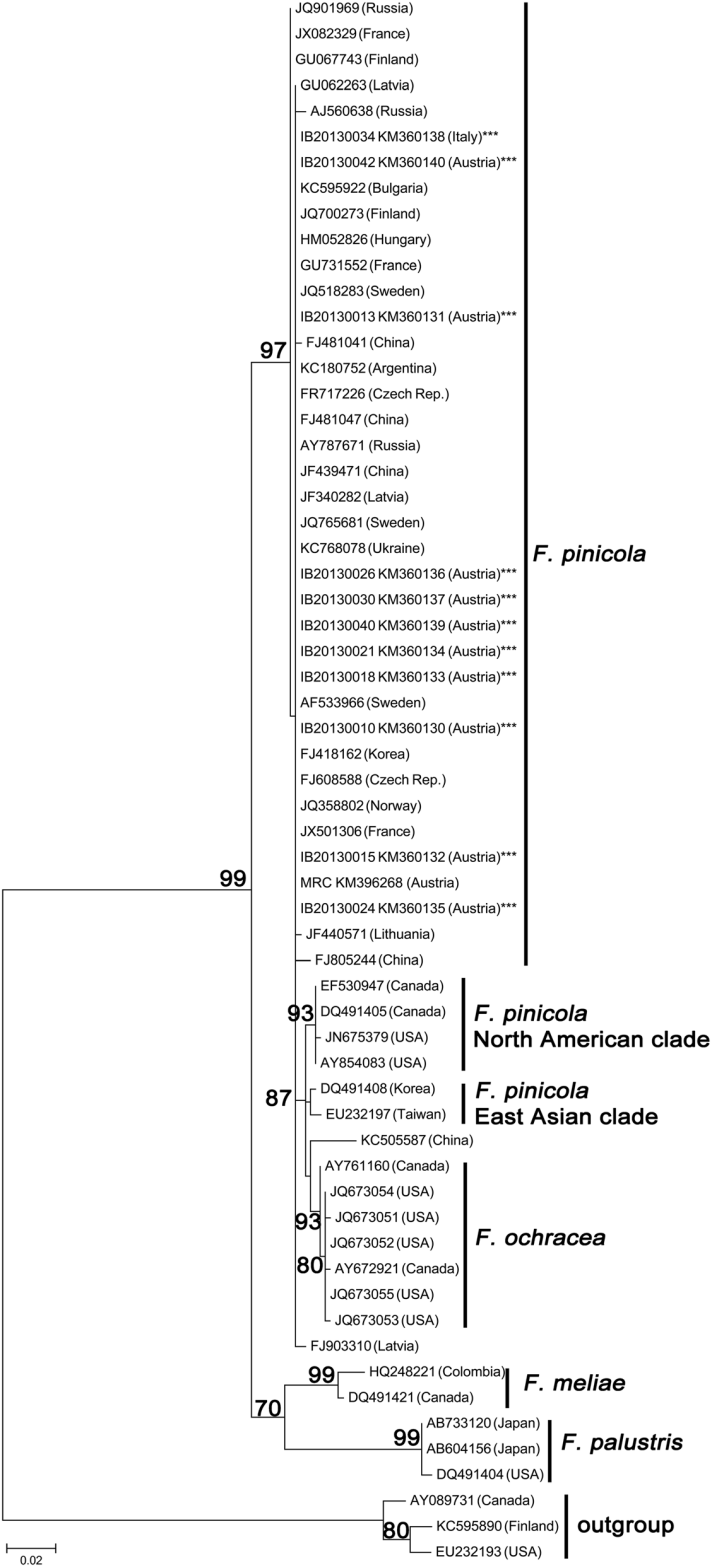
**Phylogenetic placement of** ***F. pinicola*** **strains inferred by using the Maximum Likelihood method based on the Hasegawa-Kishino-Yano model.** The tree with the highest log likelihood (-1629.3947) is shown. Initial tree(s) for the heuristic search were obtained by applying the Neighbor-Joining method to a matrix of pairwise distances estimated using the Maximum Composite Likelihood (MCL) approach. A discrete Gamma distribution was used to model evolutionary rate differences among sites (5 categories (+G, parameter = 0.3750)). The tree is drawn to scale, with branch lengths measured in the number of substitutions per site. The analysis involved 61 nucleotide sequences. All positions with less than 95% site coverage were eliminated. There were a total of 504 positions in the final dataset. Bootstrap values above 75 % are given (1 000 replications). Evolutionary analyses were conducted in MEGA6.

*F. pinicola* is a polypore with very little sequence divergence between strains. Only sequences from USA or Asia showed some sequence divergence in addition to *F. ochracea*, which forms a distinct clade within *F. pinicola,* thus making this clade paraphyletic (Figure 5)*.* We found very little sequence divergence between all our strains: Sequence divergence to other isolates of *F. pinicola* was 1-5 base pairs out of 504 positions, to other species of *Fomitopsis* (*F. meliae, F. palustris*) 15-21 base pairs.

#### Phylogenetic placement of *P. betulinus* strains

**Figure 6:**
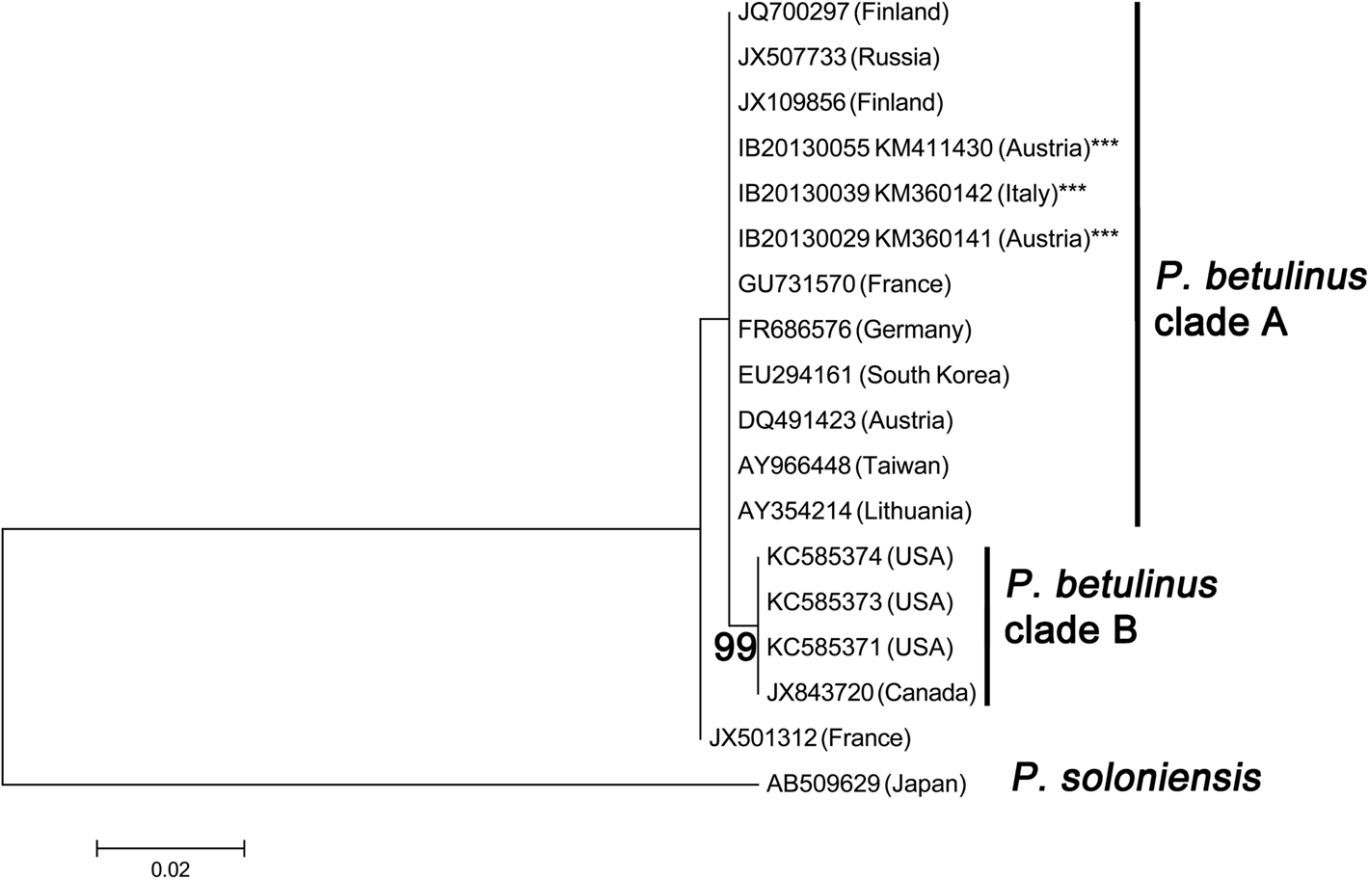
**Phylogenetic placement of** ***P. betulinus*** **strains inferred by using the Maximum Likelihood method based on the Kimura 2-parameter model.** The tree with the highest log likelihood (−529.8218) is shown. Initial tree(s) for the heuristic search were obtained by applying the Neighbor-Joining method to a matrix of pairwise distances estimated using the Maximum Composite Likelihood (MCL) approach. The tree is drawn to scale, with branch lengths measured in the number of substitutions per site. The analysis involved 18 nucleotide sequences. All positions with less than 95% site coverage were eliminated. That is, fewer than 5% alignment gaps, missing data, and ambiguous bases were allowed at any position. There were a total of 256 positions in the final dataset. Evolutionary analyses were conducted in MEGA6.

*P. betulinus* is a polypore species with little rDNA ITS sequence variation between different strains from a broad geographic area (Europe, Russia, East Asia) and *Betula* spp. or *Acer saccharum* as substrate (Figure 6): Within species sequence divergence was very low: *P. betulinus* sequences differed only in 1-2 out of > 500 base pairs from each other, but had 44-45 differed base pairs when compared to the sister taxon *P. soloniensis*.

## Discussion

### Fungal strain matters

Our results confirmed our hypothesis that medicinal polypore strains belonging to the same lineage differ significantly in their qualitative and quantitative secondary metabolite production, and in bioactivity. This confirms earlier studies focusing on other medicinal polypore species showing that the fungal strain used is important for the chemical composition of fruit bodies (Agafonova et al. 2007) and also for the production of bioactive secondary metabolites (Lv et al. 2012; Sørensen and Giese 2013). We therefore recommend that strain selection and preliminary bioactivity testing should become common practice for all studies focusing on constituents and biological effects of medicinal polypores. Fungal strain selection is routinely applied for a wide range of species used for food (Chiu et al. 1999; Liu et al. 2012; Terashima et al. 2002), biotechnology (Geiger et al. 2012), and biocontrol (Cui et al. 2014).

All strains used in this study were isolated from those fruit bodies which were later used for extraction and bioactivity testing. Their colony growth rates were tested under standard conditions in order to investigate strain-specific properties without the influence of different substrates or environmental factors. When considering only strains of *F. fomentarius* belonging to the same lineage, the quantity and quality of secondary metabolites produced by temperate strains showed clear differences. One strain isolated from *Fagus* (IB20130019) exhibited significantly different growth characteristics compared to all other strains isolated from *Picea*: It was growing significantly slower and had a lower optimal temperature. This strain was also extremely differing in metabolite production and bioactivity.

Also *F. pinicola* strains isolated from *Picea* had different growth rates and optimum temperatures than strains isolated from other substrates (*Abies*, *Alnus,* or *Larix*) even when originating from the same geographical area. Bioactivity tests confirmed these strain-specific differences, because all strains from other substrates had a stronger antifungal activity than strains isolated from *Picea*. This suggests that substrate is a strong trigger for or may influence metabolite production. In any case, the strain-specific differences remained stable under standardized conditions (*in vitro*). Additional studies comparing extracts from wild-grown fruit bodies with extracts from fruit bodies cultivated *in vitro* (under standard conditions) from the same strain will further elucidate the influence of strain characteristics on the metabolite profile and the respective bioactivities. Testing different environmental parameter and substrates is a suitable method to pre-select strains of medicinal polypores and their growth conditions for upscaling to increase the biosynthesis of bioactive secondary metabolites.

### The influence of substrate on bioactivity and metabolites from fruit bodies

Metabolite production is often substrate dependent, and selected bioactive metabolites may be produced on selected substrates only (Shang et al. 2012), or produced in significantly different quantities (Sørensen and Giese 2013). Even minor variations in environment or nutrition have the potential to affect the quantity and diversity of fungal metabolites. The deliberate elaboration of cultivation parameters to influence the secondary metabolism of a strain has been called the OSMAC (one strain, many compounds) approach (Bode et al. 2002). Substrate modifications have already been used to enhance secondary metabolite production and bioactivity of the most popular medicinal polypores, i.e. Reishi (*G. lucidum*) (You et al. 2012) and Chaga (*Inonotus obliquus*) (Xu and Zhu 2011). In *F. fomentarius,* submerged culturing processes were optimized for the production of bioactive polysaccharides (Chen et al. 2008, Chen et al. 2011) and for laccase production (Neifar et al. 2011). Up to now, we are not aware of studies focusing on the effect of substrate on the bioactivity of medicinal polypore fruit bodies. Future studies based on fruit bodies cultivated under controlled conditions and on different substrates will help to elucidate this issue.

### The medicinal potential of *F. fomentarius*, *F. pinicola* and *P. betulinus*

Our bioactivity screening generally confirms the potential of *F. fomentarius*, *F. pinicola* and *P. betulinus* as medicinal polypores. Because of their bioactivity against gram-positive bacteria, and their potency as antifungal agent, we especially consider *F. pinicola* to be worth further investigation on a molecular level. This fungus was widely applied in traditional European medicine, but its benefits and utilisation have been forgotten after the introduction of synthetic drugs. Due to renaissance of naturopathy and also due to increasing bacterial and fungal resistances, working with traditional medicinal fungi is becoming increasingly interesting and rewarding.

Most of our polypore extracts inhibited the growth of the gram-positive bacterium *S. aureus* and of the potentially pathogenic fungus *Absidia orchidis* (*Mucorales*)*;* In addition, we detected varying bioactivities (MICs of 31-1000 μg mL^−1^) for *B. subtilis*, *Aspergillus flavus, A. fumigatus* and *Candida krusei*. These varying bioactivities are either species-specific, or related to strain properties.

Crude extracts are generally a mixture of active and non-active compounds, and MICs < 100 μg mL^−1^ generally suggest good antimicrobial activity (Dellavalle et al. 2011; Webster et al. 2008). In this research, MIC values were often lower than 100 μg mL^−1^, demonstrating strong antibacterial activity of extracts under study. Antifungal activities were slightly higher with lowest values of 125 μg mL^−1^. The MIC of our *F. pinicola* extracts against *B. subtilis* (31-125 μg mL^−1^) can be considered as very good, especially when compared to MIC values as previously reported for six isolated *F. pinicola* compounds against *B. cereus* (16-128 μg mL^−1^) (Liu et al. 2010). The MICs of our *P. betulinus* extracts (31-62.5 μg mL^−1^) are also significant, but slightly higher than the values of 17.5 μg mL^−1^ achieved based on agar-well diffusion assays, as reported for Serbian *P. betulinus* extracts against antibiotic-resistant gram-positive bacteria (*Bacillus sp., Rhodococcus equi,* and *S. aureus*). We could not detect significant bioactivity against gram-negative bacteria (*Pseudomonas aeruginosa* or *Escherichia coli*), as earlier reported for a *P. betulinus* strain from Serbia (Karaman et al. 2009).

It is difficult to compare results on bioactivity of different studies, because of the different extraction methods, test organisms and test systems applied. Frequently used test systems are the agar cup method (Popova et al. 2009), the agar dilution assay (Guler et al. 2009), a TLC bioassay (Keller et al. 1996), or standard MIC assays based on the broth micro-dilution method (CLSI, 2012) as used in this study. Also *in vivo* models have been applied (Seniuk et al. 2011; Seniuk et al. 2013). Moreover, research groups use different samples in their test models, e.g. whole fungal extracts (Karaman et al. 2009; Turkoglu et al. 2007), fractions (Lemieszek et al. 2009) or single compounds (Hwang et al. 2013). Finally, species identification and documentation is often fragmentary, thus not allowing for direct comparison of results.

### Species delimitation first, then strain selection

The present study highlights the importance of exact species delimitation in medicinal polypores as a prerequisite for correct species identification. For *F. pinicola* and *P. betulinus,* the morphological species concepts widely agree with the phylogenetic species delimitation*. F. pinicola* phylograms showed little genetic differentiation, because this is a highly outcrossing heterothallic fungus, with panmictic conditions (Högberg et al. 1999). Also *P. betulinus* has very low sequence divergence between strains from geographically distant origins, e.g. Canada, Asia and Europe.

*Fomes fomentarius* can rather be considered to be a species complex (Judova et al. 2012; Pristas et al. 2013), as corroborated by our phylogenetic analysis: North European, South European, and Asian *F. fomentarius* strains fall into three distinct lineages, and representatives of each lineage are growing on different host tree species. Therefore, these lineages should be considered as distinct *Fomes* species representing different lineages of evolution. Our *F. fomentarius* strain isolated from *Quercus* from a sub-Mediterranean area (IB20130033) belongs to the well-supported South European clade, and has comparatively large sequence divergence to all our strains from the temperate area. Thus, we consider this Mediterranean *F. fomentarius* strain as a distinct taxon. This strain also differs from temperate strains by a significantly higher temperature optimum, and different growth rates, thus supporting a hypothesis suggesting that tropical species of wood-decay fungi are characterized by a higher temperature optima for growth (30-40°C) than temperate wood decay fungi (20-30°C) (Magan 2008). Growth rates have been shown to be species-specific in *Fomes* (McCormick et al. 2013), which is further supporting our hypothesis that this lineage represents a distinct species.

Species delimitation is an important issue in medicinal fungi, because the production of bioactive secondary metabolites is first species-specific, and then depending on strain characteristics. Physiological properties, e.g. growth rates and optimum temperature, are generally useful characters for species delimitation in fungi (Jaklitsch 2009) and have been applied for corticoid (Hallenberg and Larsson 1991) and poroid (McCormick et al. 2013) wood-inhabiting *Basidiomycota*. Also pigments and other metabolites are good indicators for the distinction of fungal taxa (Breheret et al. 1997; Frisvad et al. 2008). The European name *Ganoderma lucidum* (= Reishi mushroom), for example, has been widely applied on a global scale, but represents several distinct lineages or species (Wang et al. 2012), which differ in their secondary metabolite profiles (Lv et al. 2012). Also *Laetiporus sulphureus,* which is another important medicinal polypore, includes several lineages representing distinct species with different geographic origin and from different hosts (Banik et al. 2012). Ecological adaptation through host shifts and substratum specialization is likely to be an important mode of speciation and adaptive radiation in polypores (Skaven Seierstad et al. 2013). A successful screening on bioactive secondary metabolites must therefore be based on two stable pillars: first, a reliable species identification followed by an extensive screening for a suitable or ‘best strain’.

## Additional file

Additional file 1:
**HPLC chromatograms of fruit body extracts and colony growth of fungal strains under different temperatures.**
