# Supplementary material for: Fungal strain matters: colony growth and bioactivity of the European medicinal polypores Fomes fomentarius, Fomitopsis pinicola and Piptoporus betulinus
Source: AMB Express. 2015 Jan 24;5:4. doi: 10.1186/s13568-014-0093-0 (PMC4305089; doi:10.1186/s13568-014-0093-0)
Supplement: Additional file 1: — HPLC chromatograms of fruit body extracts and colony growth of fungal strains under different temperatures. [file 13568_2014_93_MOESM1_ESM.pdf]

Fungal strain matters: Colony growth and bioactivity of the European medicinal polypores *Fomes fomentarius, Fomitopsis pinicola* and *Piptoporus betulinus*

AMB Express

Philipp Dresch, Maria Nives D´Aguanno, Katharina Rosam, Ulrike Grienke, Judith Rollinger, Ursula Peintner*

*Institute of Microbiology, University of Innsbruck, Technikerstraße 25, 6020 Innsbruck, Austria, E-mail: ursula.peintner@uibk.ac.at

Supplementary Material

Fig. S1 – HPLC chromatograms of ethanolic polypore fruit body extracts. Analyses were carried out using a Shimadzu UFLC XR device at 40 °C, a flow rate of 1.0 mL min^{-1}, injection volume was 10 µL, detection wavelength 254 nm. The stationary phase was a Phenomenex® HyperClone ODS (C18) column, 120 Å, 5µm, 150 x 4.60 mm; mobile phase A: H2O + 0.9 % AA + 0.1 % FA; mobile phase B: ACN + 0.9 % AA + 0.1 % FA; 0 min: 90 % A to 30 min: 2 % A, 30-45 min: 2 % A.

Fomes fomentarius

Fomfom IB20130011

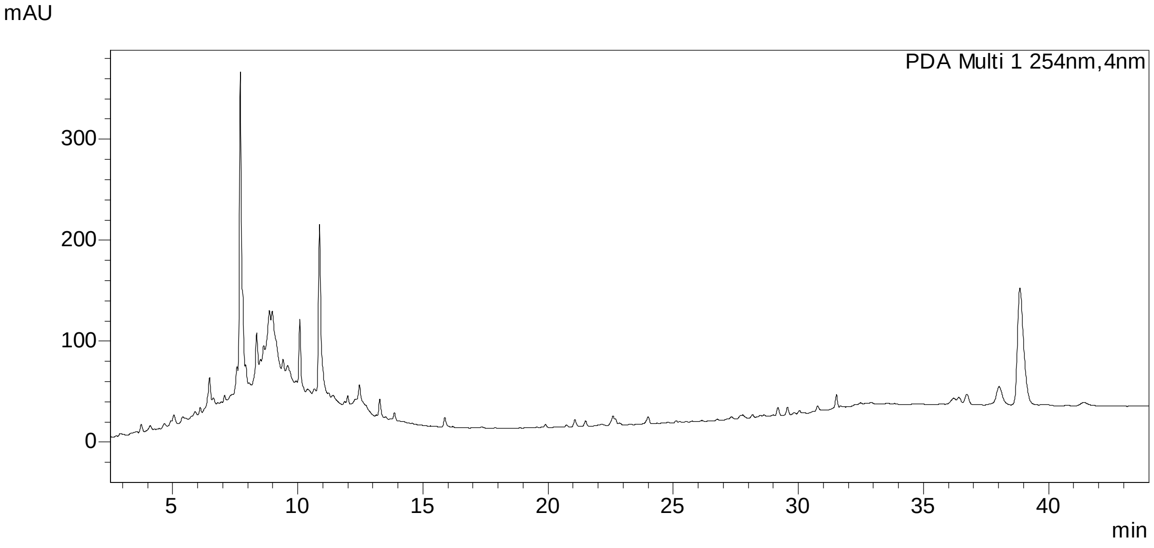

Fomfom IB20130016

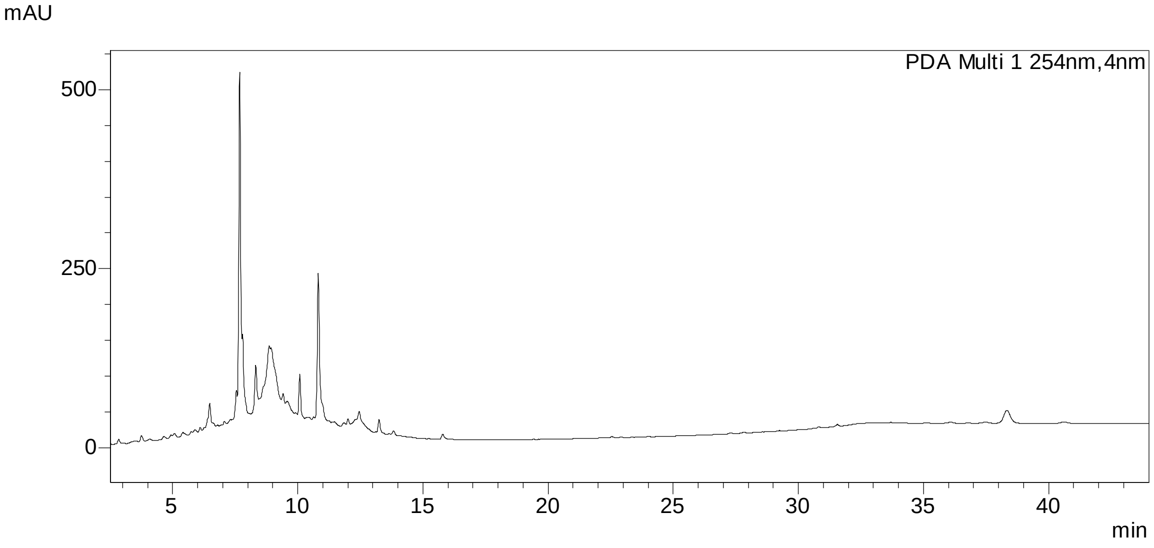

Fomfom IB20130019

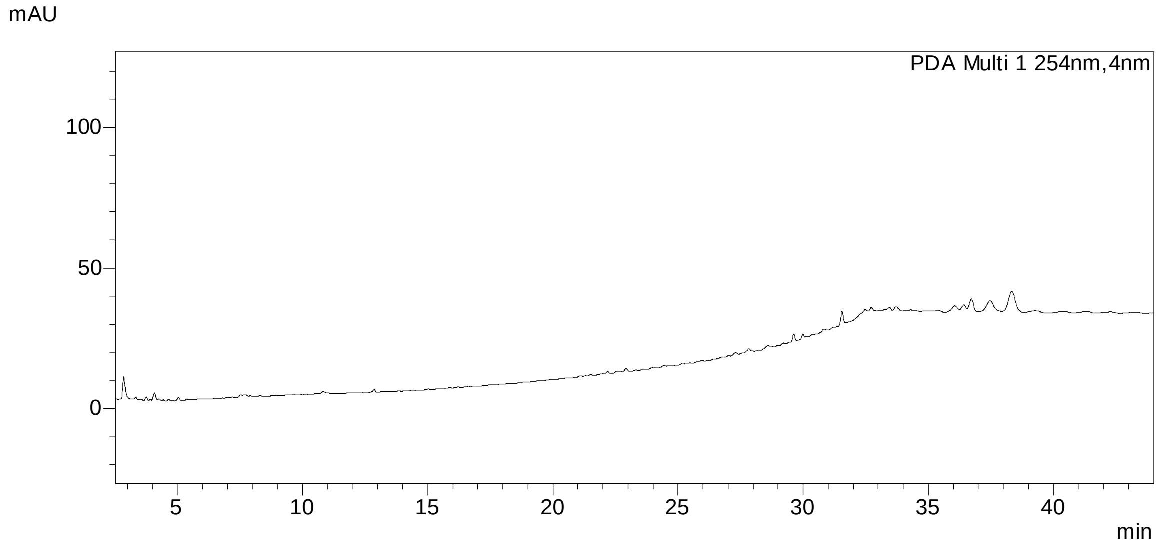

Fomfom IB20130022

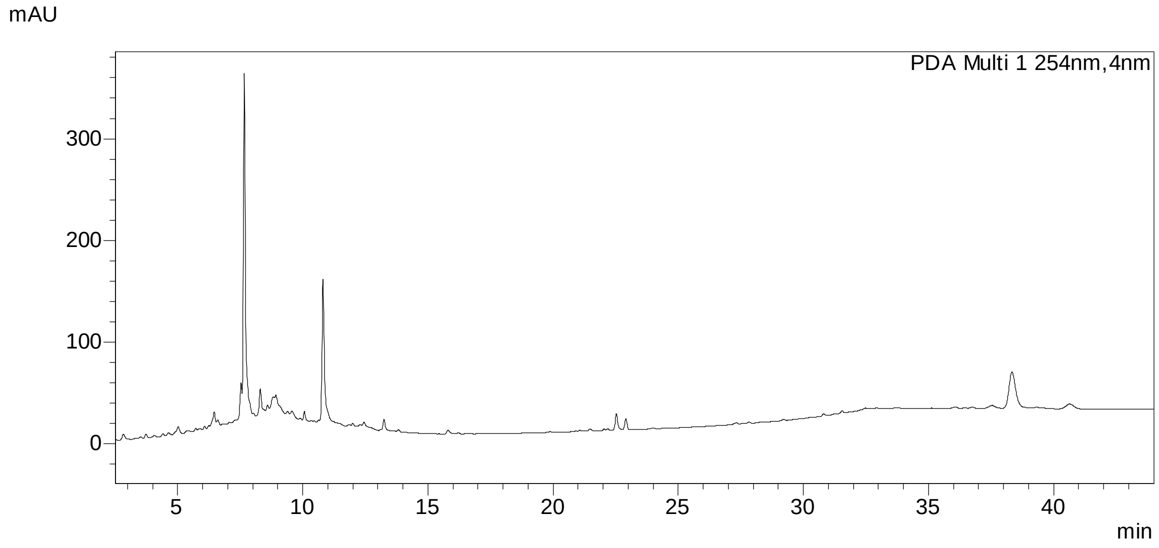

Fomfom IB20130033

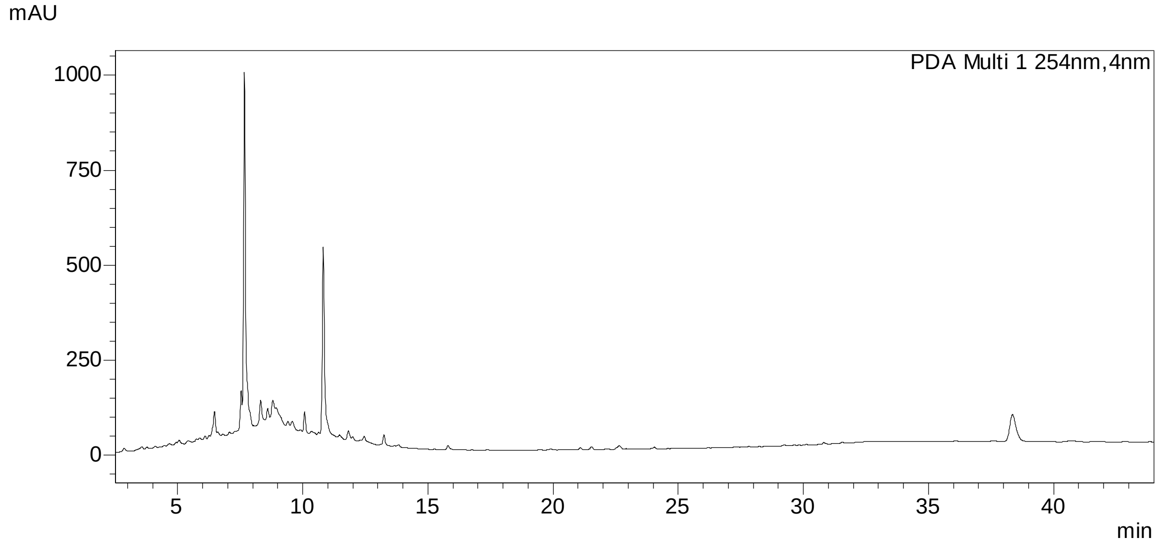

Fomitopsis pinicola

Fompin IB20130010

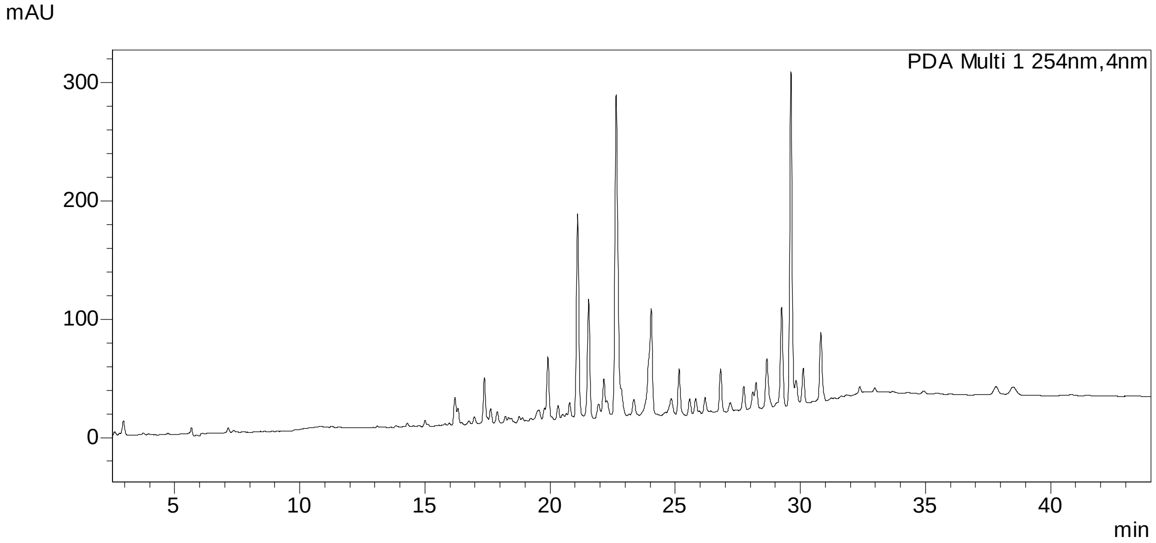

Fompin IB20130013

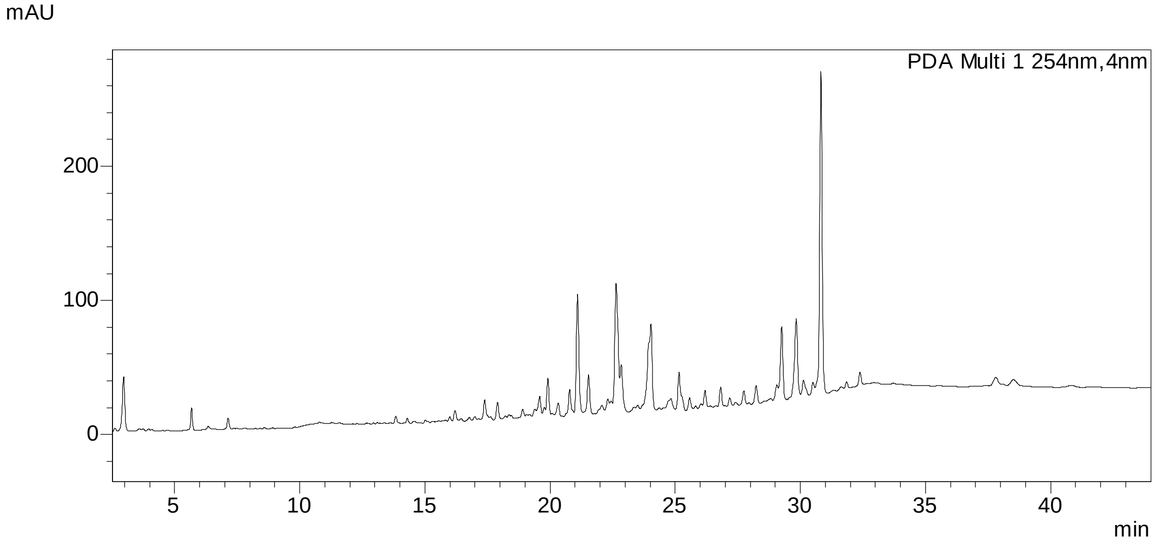

Fompin IB20130015

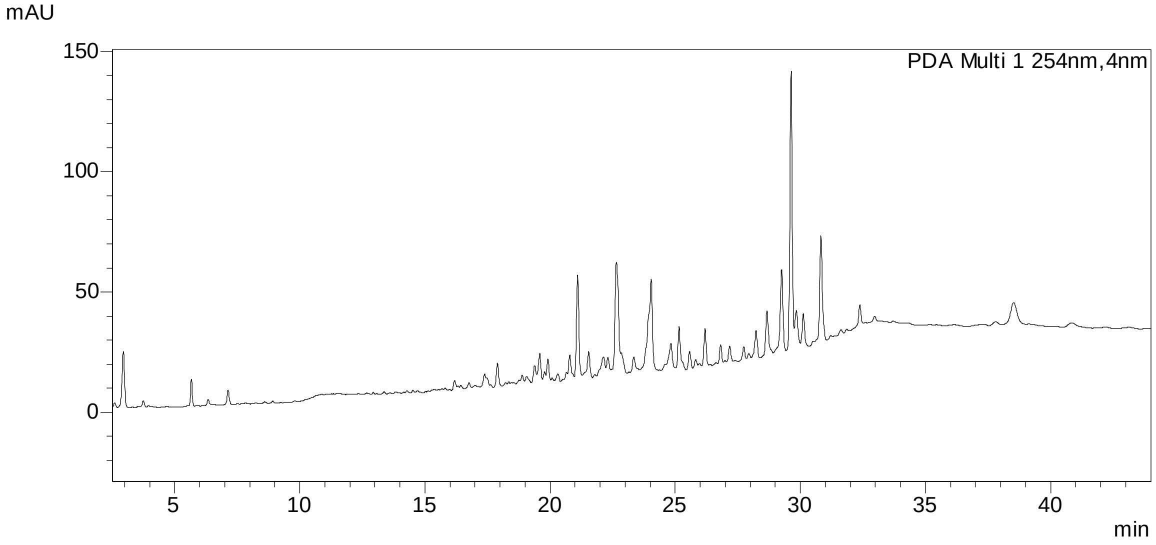

Fompin IB20130018

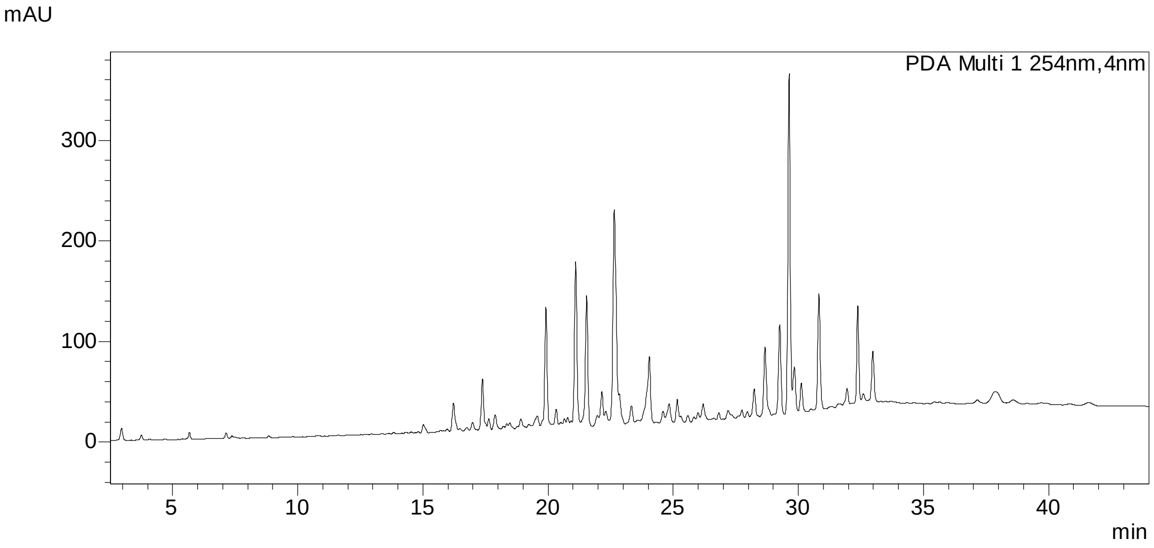

Fompin IB20130021

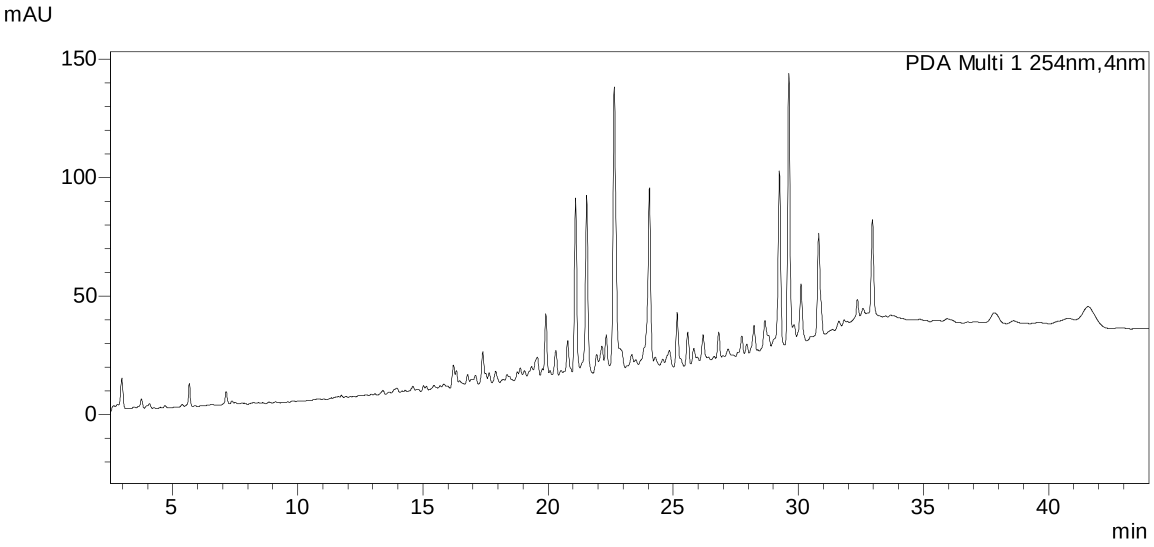

Fompin IB20130024

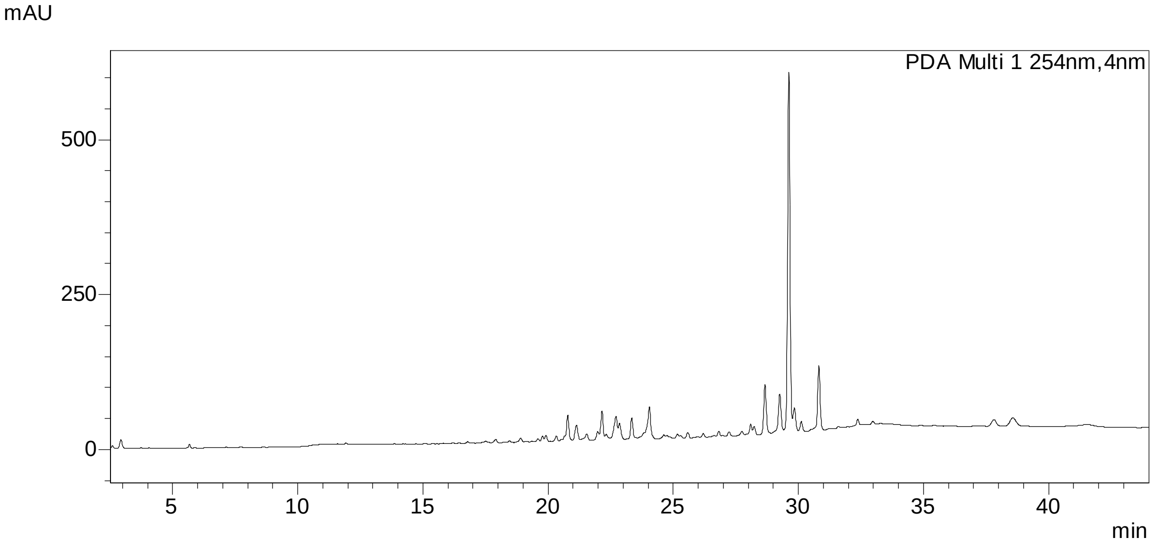

Fompin IB20130026

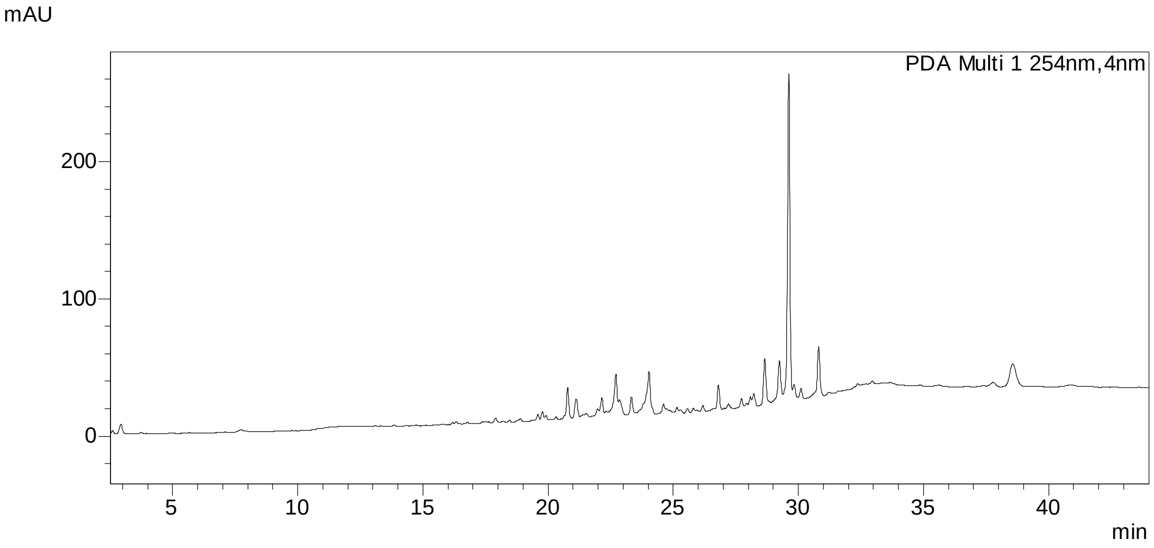

Fompin IB20130030

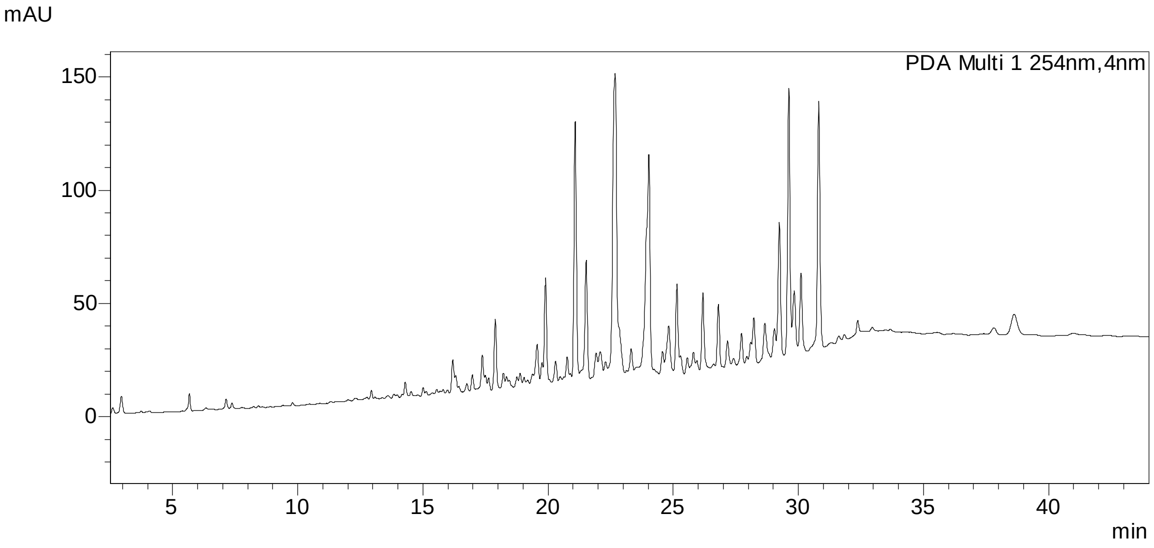

Fompin IB20130037

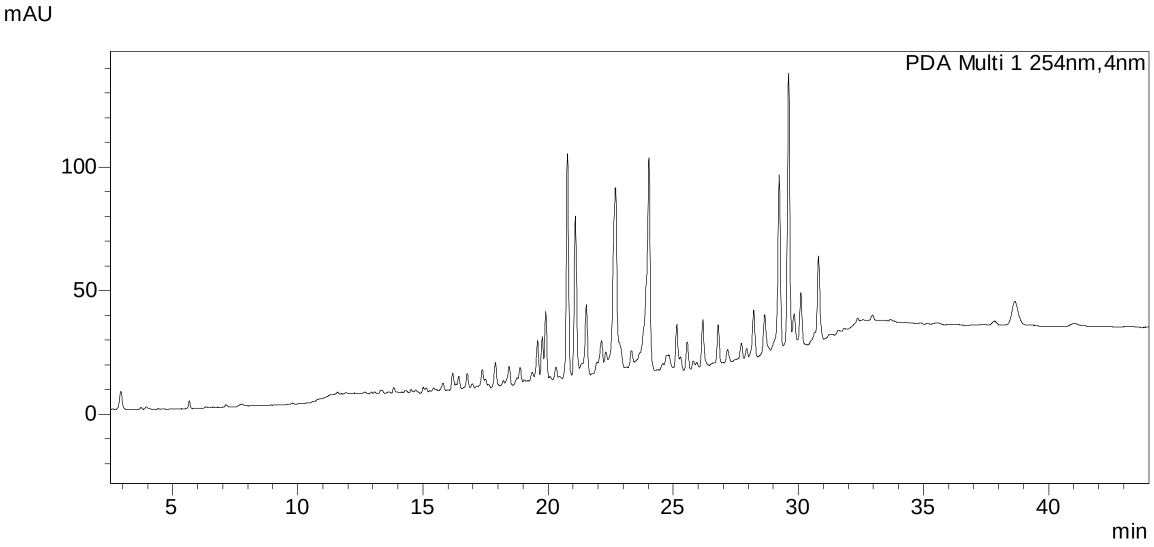

Fompin IB20130040

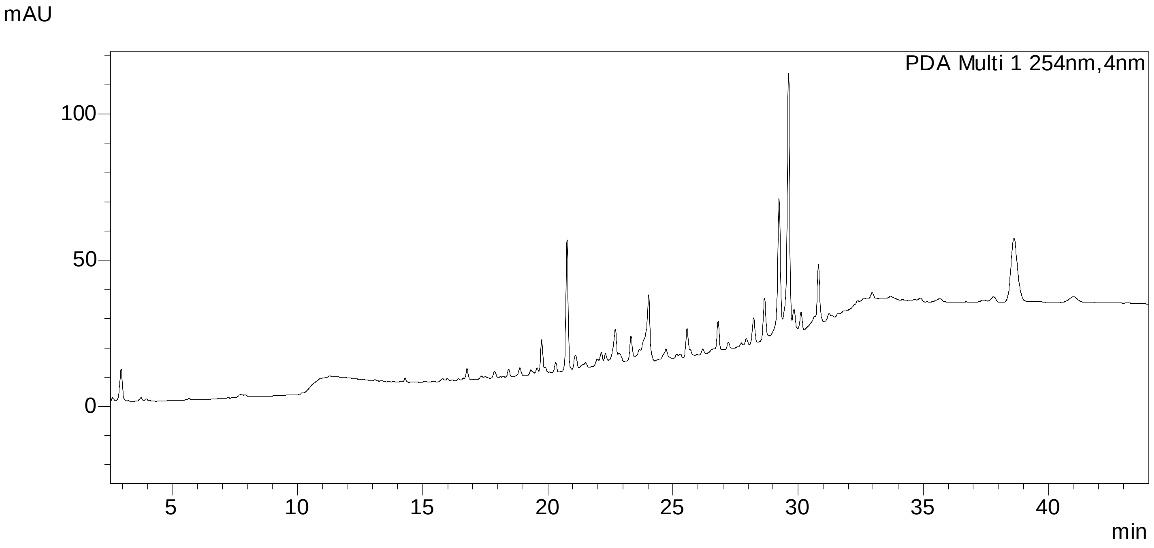

Fompin IB20130053

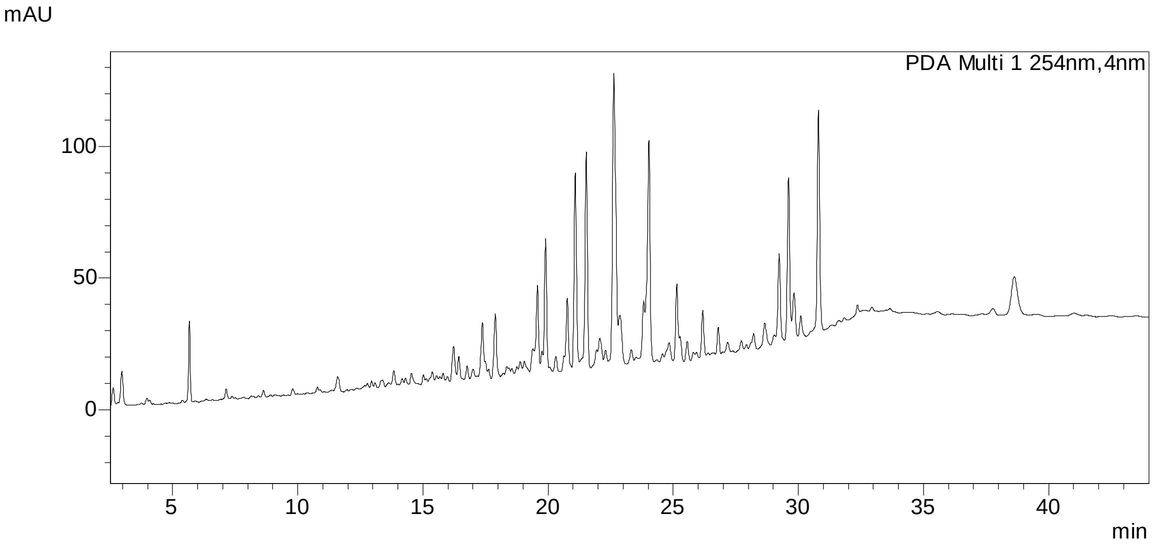

Fompin IB20130034

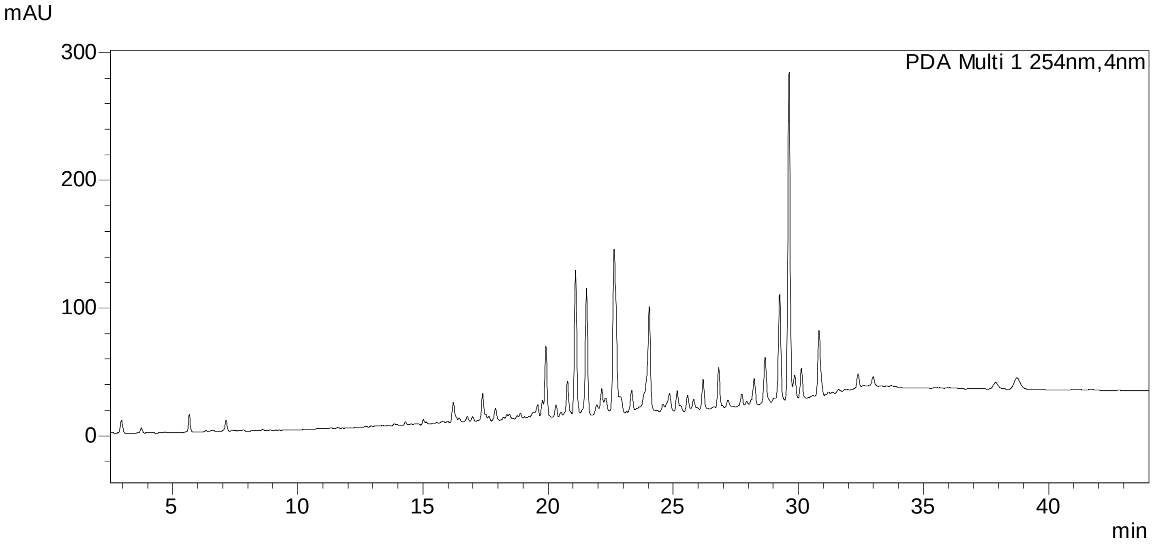

Fompin IB20130042

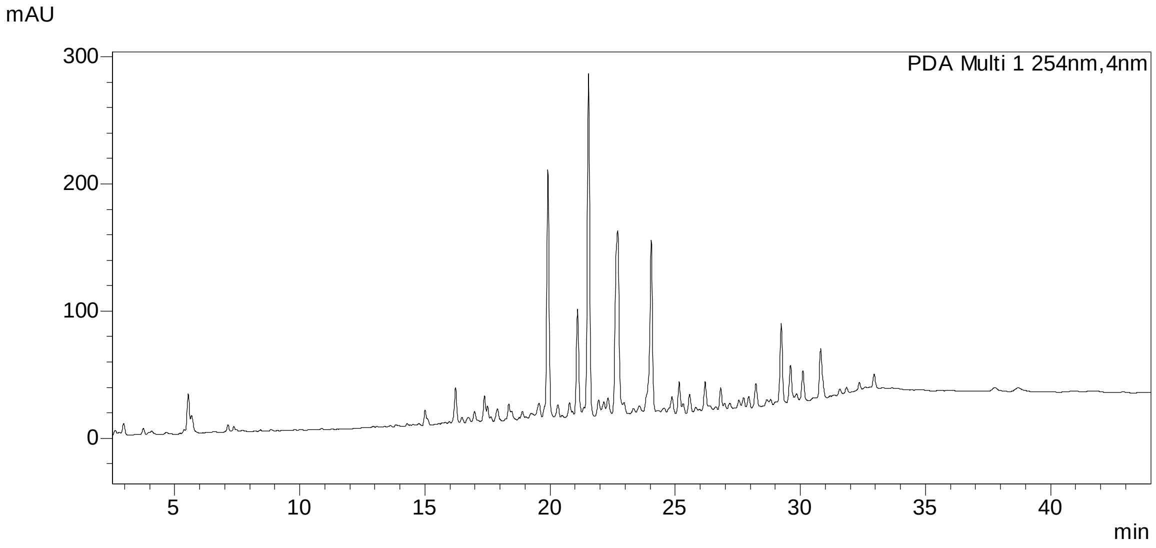

Piptoporus betulinus

Pipbet IB20130029

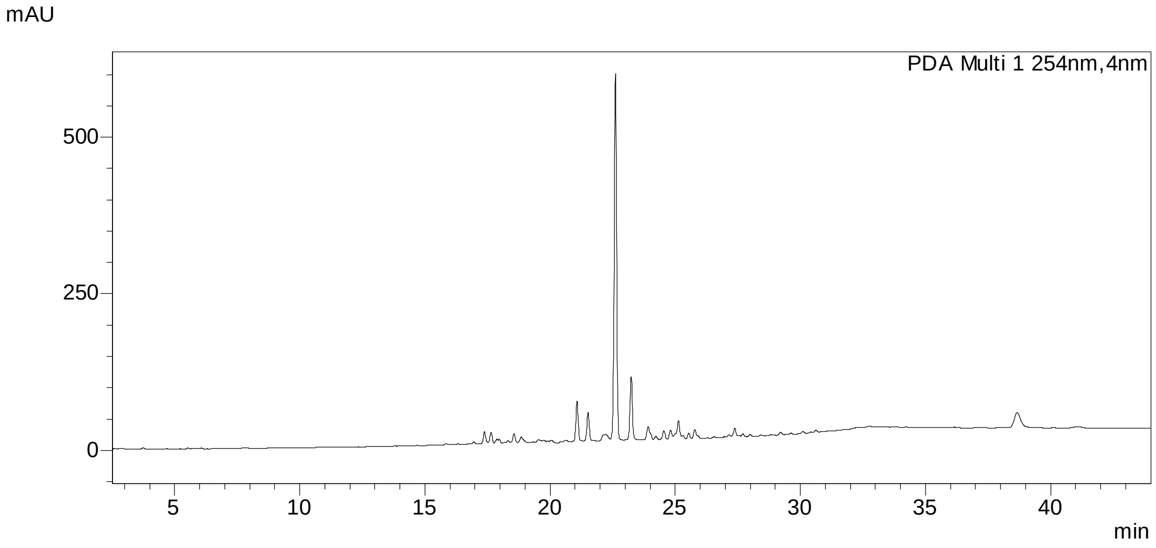

Pipbet IB20130039

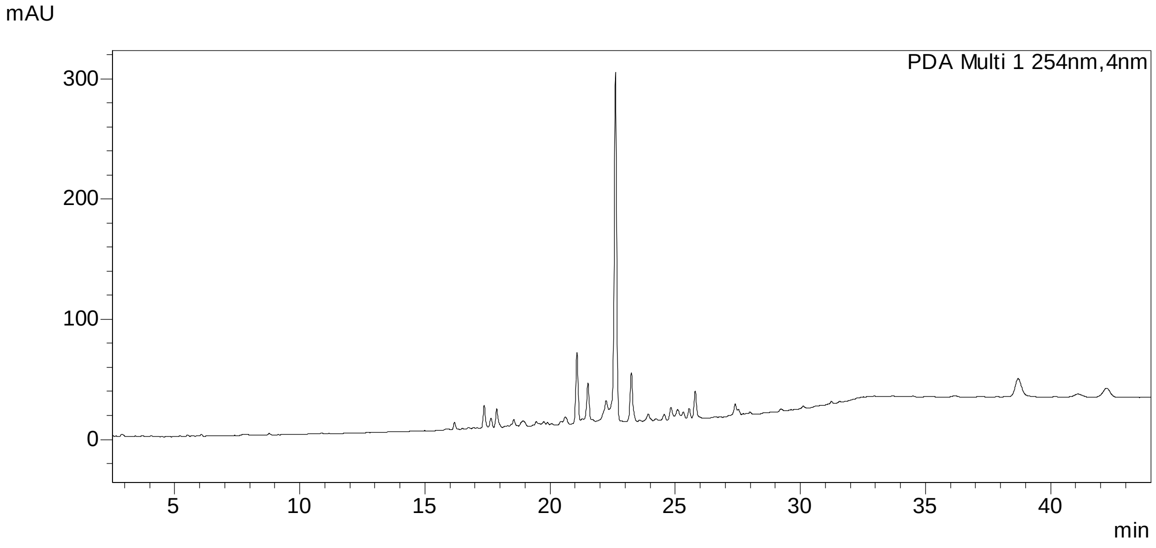

Table S1 - Mean colony diameter and standard deviation of isolated *Fomes fomentarius* strains grown under different temperatures measured over a course of 10 days (n = 3).

Temperature	strain	2 days		5 days		7 days		10 days	
		Mean	SD	Mean	SD	Mean	SD	Mean	SD
**10 °C**	20130011	0.00	0.00	0.83	0.15	0.13	0.06	0.83	0.15
	20130016	0.00	0.00	0.13	0.06	0.13	0.06	0.83	0.06
	20130019	0.00	0.00	0.13	0.06	0.07	0.06	1.23	0.31
	20130022	0.30	0.10	1.23	0.06	1.27	0.25	1.53	0.12
	20130033	0.00	0.00	0.00	0.00	0.53	0.15	0.70	0.20
**20 °C**	20130011	0.00	0.00	2.70	0.26	2.93	0.23	4.60	3.58
	20130016	0.00	0.00	4.67	0.29	4.83	0.32	7.10	0.10
	20130019	0.00	0.00	1.43	0.49	1.73	0.51	7.03	0.32
	20130022	1.17	0.06	3.07	0.15	4.50	0.46	6.13	0.47
	20130033	0.00	0.00	0.47	0.06	3.23	0.15	6.17	0.21
**25 °C**	20130011	0.13	0.02	4.13	0.80	7.13	0.80	8.80	0.26
	20130016	0.16	0.05	5.93	0.72	7.63	0.64	8.70	0.30
	20130019	0.13	0.01	2.87	0.45	5.07	0.42	7.90	0.30
	20130022	0.14	0.01	4.77	0.61	8.13	0.21	8.90	0.17
	20130033	0.93	0.15	2.73	0.57	4.07	0.47	6.43	0.50
**30 °C**	20130011	0.93	0.40	6.83	1.07	7.07	1.27	8.57	0.06
	20130016	1.07	0.06	7.13	0.15	7.40	0.20	8.57	0.06
	20130019	0.43	0.12	2.17	0.06	2.43	0.06	8.60	0.00
	20130022	1.60	0.10	6.23	0.12	8.13	0.21	8.53	0.06
	20130033	0.13	0.06	3.47	0.31	5.60	2.87	8.30	0.46
**32 °C**	20130011	0.39	0.25	5.40	1.15	5.33	0.23	6.63	0.45
	20130016	0.28	0.18	3.90	0.61	5.13	0.25	6.73	0.25
	20130019	0.03	0.06	3.47	0.47	3.77	0.51	5.40	0.53
	20130022	0.12	0.02	4.64	0.34	4.97	0.25	7.03	0.23
	20130033	1.31	0.12	6.23	0.49	8.67	0.49	8.97	0.06
**37 °C**	20130011	0.00	0.00	0.00	0.00	0.00	0.00	0.00	0.00
	20130016	0.00	0.00	0.00	0.00	0.00	0.00	0.00	0.00
	20130019	0.00	0.00	0.00	0.00	0.00	0.00	0.00	0.00
	20130022	0.17	0.12	0.23	0.06	1.07	0.15	1.53	0.06
	20130033	0.00	0.00	0.00	0.00	0.00	0.00	0.00	0.00

Table S2 - Mean colony diameter and standard deviation of isolated *Fomitopsis pinicola* strains grown under different temperatures measured over a course of 10 days (n = 3).

Temperature	strain	2 days		5 days		7 days		10 days	
		Mean	SD	Mean	SD	Mean	SD	Mean	SD
**10 °C**	20130010	0.00	0.00	0.07	0.06	0.13	0.06	1.70	0.35
	20130013	0.00	0.00	0.13	0.06	0.17	0.06	1.43	0.15
	20130015	0.00	0.00	0.17	0.06	0.23	0.06	1.67	0.31
	20130018	0.00	0.00	0.23	0.06	0.23	0.12	2.77	1.51
	20130021	0.00	0.00	0.23	0.12	0.13	0.06	1.70	0.26
	20130026	0.60	0.10	1.10	0.10	1.07	0.21	1.47	0.15
	20130030	0.00	0.00	0.40	0.10	1.03	0.15	1.30	0.20
	20130034	0.10	0.10	0.33	0.21	0.43	0.12	1.90	0.26
	20130040	0.13	0.06	0.43	0.06	0.20	0.17	0.40	0.69
	20130042	0.00	0.00	0.13	0.06	0.20	0.10	1.37	0.12
**20 °C**	20130010	0.00	0.00	6.03	0.32	6.27	1.45	7.87	0.32
	20130013	0.00	0.00	6.17	0.31	6.47	0.31	7.80	0.26
	20130015	0.00	0.00	5.83	0.15	6.03	0.15	8.03	0.06
	20130018	0.00	0.00	4.50	0.20	4.70	0.20	3.00	0.69
	20130021	0.00	0.00	4.83	0.32	5.03	0.32	7.37	0.47
	20130026	1.20	0.10	3.50	0.10	5.27	0.25	6.83	0.15
	20130030	0.13	0.06	1.83	0.25	2.27	0.49	2.73	0.38
	20130034	0.13	0.06	1.77	0.15	5.00	0.26	7.10	0.26
	20130040	0.23	0.06	1.70	0.17	0.90	0.10	1.27	0.15
	20130042	0.00	0.00	1.00	0.10	5.90	0.10	7.93	0.15
**25 °C**	20130010	0.42	0.20	6.77	0.21	7.70	1.04	8.93	0.06
	20130013	0.37	0.31	6.47	0.31	7.30	0.70	8.20	0.62
	20130015	0.20	0.10	6.13	0.15	8.53	0.91	8.80	0.35
	20130018	0.13	0.15	6.17	0.20	7.10	0.53	8.27	0.55
	20130021	0.03	0.06	6.97	0.32	7.13	0.80	8.37	0.60
	20130026	0.13	0.15	5.03	0.10	5.37	0.15	6.03	0.45
	20130030	1.44	0.34	4.13	0.25	4.83	0.21	6.00	1.01
	20130034	2.02	0.48	4.80	0.15	6.67	0.87	7.43	0.55
	20130040	2.94	0.75	7.63	0.17	8.17	0.23	8.57	0.45
	20130042	0.67	0.35	6.90	0.10	7.63	1.19	8.97	0.06
**30 °C**	20130010	1.17	0.44	6.57	0.21	6.77	0.21	8.57	0.06
	20130013	1.13	0.15	6.57	0.40	6.67	0.58	8.47	0.06
	20130015	1.10	0.10	5.23	0.21	5.43	0.21	8.57	0.06
	20130018	1.47	0.15	6.93	0.12	7.13	0.12	8.60	0.00
	20130021	1.50	0.00	6.97	0.15	7.17	0.15	8.53	0.06
	20130026	1.57	0.15	5.03	0.21	6.77	0.15	7.80	0.10
	20130030	1.00	0.20	2.40	0.40	8.07	0.21	8.30	0.46
	20130034	1.17	0.31	2.80	0.66	2.00	0.10	8.90	0.00
	20130040	0.20	0.10	1.27	0.25	6.60	0.40	2.43	4.21
	20130042	1.13	0.15	1.03	0.06	6.90	0.10	8.57	0.06

Continuation Table S2

**32 °C**	20130010	0.45	0.45	4.40	0.53	5.53	0.58	6.43	0.10
	20130013	1.65	0.44	3.93	0.06	4.87	0.32	5.57	0.46
	20130015	2.10	0.69	4.53	1.10	5.07	0.96	5.83	0.81
	20130018	3.04	0.61	4.60	0.61	5.43	1.08	6.63	0.38
	20130021	0.00	0.00	3.83	1.21	6.47	0.49	6.83	0.67
	20130026	2.75	0.52	6.87	0.15	8.43	0.49	8.97	0.58
	20130030	3.21	0.10	7.00	0.10	8.57	0.67	8.90	0.32
	20130034	0.50	0.30	1.03	0.15	1.73	0.29	5.40	0.96
	20130040	2.18	0.34	4.87	0.32	5.93	0.86	6.63	1.08
	20130042	1.22	0.23	4.67	0.59	5.17	0.91	6.17	0.49
**37 °C**	20130010	0.00	0.00	0.00	0.00	0.00	0.00	0.00	0.00
	20130013	0.00	0.00	0.00	0.00	0.00	0.00	0.00	0.00
	20130015	0.00	0.00	0.00	0.00	0.00	0.00	0.00	0.00
	20130018	0.00	0.00	0.00	0.00	0.00	0.00	0.00	0.00
	20130021	0.00	0.00	0.00	0.00	0.00	0.00	0.00	0.00
	20130026	0.97	0.21	0.20	0.10	1.27	0.12	1.47	0.12
	20130030	0.00	0.00	0.13	0.15	0.00	0.00	0.00	0.00
	20130034	0.07	0.06	0.23	0.06	0.00	0.00	0.00	0.00
	20130040	0.00	0.00	0.00	0.00	0.00	0.00	0.10	0.17
	20130042	0.00	0.00	0.10	0.17	0.00	0.00	0.00	0.00

Table S3 - Mean colony diameter and standard deviation of isolated *Piptoporus betulinus* strains grown under different temperatures measured over a course of 10 days (n = 3).

Temperature	strain	2 days		5 days		7 days		10 days	
		Mean	SD	Mean	SD	Mean	SD	Mean	SD
**10 °C**	20130029	0.03	0.06	0.30	0.10	1.73	0.15	1.90	0.26
	20130039	0.17	0.06	0.23	0.06	0.10	0.10	1.73	0.15
	20130055	0.30	0.10	0.47	0.15	1.57	0.06	0.70	0.20
**20 °C**	20130029	1.03	0.15	2.97	0.15	5.27	0.15	7.33	0.21
	20130039	0.93	0.15	2.20	0.26	3.70	0.53	6.20	0.56
	20130055	0.47	0.15	1.67	0.61	3.67	1.01	6.30	1.04
**25 °C**	20130029	1.83	0.42	4.70	0.15	7.67	0.15	8.10	0.85
	20130039	1.02	0.11	5.67	0.26	7.30	0.53	8.07	0.90
	20130055	1.54	0.40	5.60	0.61	7.07	1.01	8.97	0.06
**30 °C**	20130029	0.87	0.25	2.70	0.44	5.37	0.40	8.33	0.21
	20130039	1.03	0.31	2.07	0.15	4.33	0.84	6.33	0.56
	20130055	0.33	0.06	1.63	0.32	5.17	0.46	7.83	1.04
**32 °C**	20130029	0.83	0.06	1.53	0.44	4.90	0.62	7.37	0.21
	20130039	0.59	0.09	2.23	0.15	3.90	2.27	6.77	0.56
	20130055	0.28	0.07	1.90	0.32	4.77	1.58	8.40	1.04
**37 °C**	20130029	0.00	0.00	0.00	0.00	0.00	0.00	0.00	0.00
	20130039	0.00	0.00	0.13	0.06	0.00	0.00	0.00	0.00
	20130055	0.07	0.06	0.07	0.06	0.13	0.06	0.13	0.06
